# A Meta-Analysis of Seaweed Impacts on Seagrasses: Generalities and Knowledge Gaps

**DOI:** 10.1371/journal.pone.0028595

**Published:** 2012-01-10

**Authors:** Mads S. Thomsen, Thomas Wernberg, Aschwin H. Engelen, Fernando Tuya, Mat A. Vanderklift, Marianne Holmer, Karen J. McGlathery, Francisco Arenas, Jonne Kotta, Brian R. Silliman

**Affiliations:** 1 Oceans Institute and School of Plant Biology, University of Western Australia, Crawley, Western Australia, Australia; 2 Centre for Marine Ecosystems Research, Edith Cowan University, Joondalup, Western Australia, Australia; 3 Australian Institute of Marine Science, Crawley, Western Australia, Australia; 4 Centro de Ciências do Mar do Algarve, Universidade do Algarve, Faro, Portugal; 5 Biodiversity and Environmental Management Centre, Universidad de Las Palmas de Gran Canaria, Las Palmas, Canary Islands, Spain; 6 The Commonwealth Scientific and Industrial Research Organisation Marine and Atmospheric Research, Wembley, Western Australia, Australia; 7 Institute of Biology, University of Southern Denmark, Odense, Denmark; 8 Department of Environmental Sciences, University of Virginia, Charlottesville, Virginia, United States of America; 9 Laboratory of Coastal Biodiversity, Centro Interdisciplinar de Investigacao Marinha e Ambiental, University of Porto, Porto, Portugal; 10 Estonian Marine Institute, University of Tartu, Tallinn, Estonia; 11 Department of Biology, University of Florida, Gainesville, Florida, United States of America; Swansea University, United Kingdom

## Abstract

Seagrasses are important habitat-formers and ecosystem engineers that are under threat from bloom-forming seaweeds. These seaweeds have been suggested to outcompete the seagrasses, particularly when facilitated by eutrophication, causing regime shifts where green meadows and clear waters are replaced with unstable sediments, turbid waters, hypoxia, and poor habitat conditions for fishes and invertebrates. Understanding the situations under which seaweeds impact seagrasses on local patch scales can help proactive management and prevent losses at greater scales. Here, we provide a quantitative review of available published manipulative experiments (all conducted at the patch-scale), to test which attributes of seaweeds and seagrasses (e.g., their abundances, sizes, morphology, taxonomy, attachment type, or origin) influence impacts. Weighted and unweighted meta-analyses (Hedges *d* metric) of 59 experiments showed generally high variability in attribute-impact relationships. Our main significant findings were that (a) abundant seaweeds had stronger negative impacts on seagrasses than sparse seaweeds, (b) unattached and epiphytic seaweeds had stronger impacts than ‘rooted’ seaweeds, and (c) small seagrass species were more susceptible than larger species. Findings (a) and (c) were rather intuitive. It was more surprising that ‘rooted’ seaweeds had comparatively small impacts, particularly given that this category included the infamous invasive *Caulerpa* species. This result may reflect that seaweed biomass and/or shading and metabolic by-products like anoxia and sulphides could be lower for rooted seaweeds. In conclusion, our results represent simple and robust first-order generalities about seaweed impacts on seagrasses. This review also documented a limited number of primary studies. We therefore identified major knowledge gaps that need to be addressed before general predictive models on seaweed-seagrass interactions can be build, in order to effectively protect seagrass habitats from detrimental competition from seaweeds.

## Introduction

Seagrasses are ubiquitous coastal plants in many tropical to cold water regions [1], [2]. Seagrasses increase habitat complexity, attenuate waves to protect coastlines, stabilize sediments, filter terrestrial run-off, bind and sequester carbon and nutrients, and provide food and shelter for invertebrates and fishes [1], [2]. These ecosystem services are currently diminishing as seagrass beds are in rapid decline around the world [1], [3], [4]. Conservation and active management of seagrass beds is therefore becoming increasingly important [5]. Current anthropogenic threats to seagrass beds include destruction of, and alterations to, coastal habitats, climate change including sea level rise and global warming, invasion by non-native species, enhanced sedimentation, and nutrient pollution [1], [3], [4], [6]. In many cases, these threats cause increasing growth of, and therefore competition from, seaweeds (macroalgae), which accelerate the degradation of seagrass habitats [7], [8], [9]. Thus, seaweeds have increasingly been implicated in the destruction of seagrass beds, particularly where nutrient pollution is high [9], [10], where fishing has reduced top-down control of seaweeds [11], or where invasive seaweeds have been introduced [12], [13]. In these cases, small patches of seaweeds can proliferate into massive mats, and ultimately convert stable seagrass meadows into less stable seaweed beds [14], [15]. During such ‘regime shifts’, habitats that are characterized by sediment stability, high water transparency, an oxic water-column, and stable standing crop, productivity and nursery function, can be replaced by habitats characterized by sediment instability, turbid waters, localized hypoxia, and greatly fluctuating macrophyte biomass, productivity and nursery function [9].

A high-priority goal in coastal zones around the world is to retain intact seagrass beds and the ecosystem services they provide [1], [3], [4], [5], [6], [8], [9]. To avoid regional regime shifts, which are difficult to manage and reverse, it is important to identify if and how *small scale seaweed patches* (< a few m^2^, reflecting the initial seaweed accumulation) impact seagrasses *before* irreversible large-scale losses occur on entire meadows. Manipulative experiments are particularly useful to address this small-scale impact issue. Manipulative experiments are also important to supplement mensurative studies that document subsequent larger-scale impacts, but provide poor mechanistic insights and may even identify misleading correlations (e.g. positive correlations between seaweed and seagrass abundances caused by physical entrapment of seaweed by the seagrass leaves) [16]. During the last few decades, a growing number of experimental case studies have documented impacts of seaweeds on seagrasses at the patch-scale (Appendix S1), but effects have varied greatly depending on the spatio-temporal and biogeographical context. For example, Hauxwell et al. [15] documented detrimental effects, whereas Ceccherelli et al. [17] found no effects, of seaweeds on seagrasses. Such discrepancies have hindered the development of a general predictive framework, and could make it more difficult to manage scenarios of increasing seagrass stress from seaweeds, for example, where coastal areas experience rapid urbanization. A few reviews have discussed general mechanisms whereby seaweeds impact seagrasses [8], [9], [18], but these qualitative approaches have no standardized methodology to compare and rank impacts between studies, seaweeds and impacted seagrasses. Meta-analysis provides a statistically rigorous method to compare impacts quantitatively across disparate studies, and thereby identify if generalities of impacts exist over and beyond the large variability that characterize ecological experiments [19], [20].

We collated experiments that tested for effects of seaweeds on seagrasses to identify which attributes might explain impacts. We aimed to provide background information for managers and scientists to approach growing problems of seaweed proliferation in coastal and estuarine areas. More specifically, based on a meta-analysis of seaweed impact experiments, we tested if the direction and magnitude of impact depended on key attributes of the seaweeds and/or seagrasses, including their abundance, size, origin, attachment type, morphology, and taxonomy. The identification of impact attributes that are simple to identify *in situ*, and that are important across studies and biogeographical regions provides a starting point for scientists and managers to address a particular bloom in progress, and from where to build advanced context-dependent models. This review also provided an opportunity to identify key research gaps in studies of seaweed impacts on seagrasses.

## Methods

The protocol for this trial and supporting CONSORT checklist are available as supporting information; see Checklist S1 and Diagram S1. We generally followed the procedures used in many ecological meta-analyses [20], [21], [22], [23], [24]. We located published experiments, where the abundance of seaweeds was manipulated to test for impacts on seagrasses, by searching in ISI Web of Science and Current Contents using various combinations of key words like ‘experiment*’, ‘seaweed*’, ‘macroalga*’, ‘epiphyt*’, ‘drift alga*’, ‘effects of’, ‘blooms’, ‘mats’, ‘*Caulerpa*’, and ‘seagrass*’ in ‘title’ and ‘abstract’ sections. We also identified relevant experiments by back-tracking references in previous reviews [e.g.], [ 8], [9,22]. We read >400 abstracts from potentially relevant papers. However, only 22 published papers (4 of which were our own) *reported seaweed impacts on seagrasses from manipulative experiments* (Appendix S1). We consider this to be a near-exhaustive list of studies describing seaweed impacts on seagrasses. The 22 studies reported impacts on seagrasses in 59 experiments. For each experiment we extracted information about attributes associated with the seaweeds and the seagrasses that potentially could influence the impact [21]. For the seaweeds, we extracted data on (1) abundance (dry weight per area; in some cases wet weight or frond density was converted to dry weight using conversion ratios; a few experiments did not report any abundance and were therefore excluded from this analysis), (2) experimental duration (months), (3) experimental plot-size (m^2^; note that duration and plot size can be considered simple proxies, at least in press-type experiments, for the temporal and spatial extent of seaweed associated stress), (4) origin (native *vs.* non-native), (5) attachment mode (unattached/drift-seaweed *vs.* rooted with rhizoids and stolons in the sediment *vs.* epiphytic attached with holdfasts to seagrass), (6) morphology, following Littler [25], except *Caulerpa* spp. were not included in this classification; rather, we treated their unique modular morphology and coenocytic cell structure as a separate morphological category, and (7) taxonomic identity (here genus). Of the 59 experiments, 17 tested specifically for impacts of seaweed abundance, i.e. they applied at least two levels of seaweed abundances [26]. From this subset of experiments, we could conduct a more detailed abundance-test, only using the data published specifically to test this impact attribute (i.e., this test does not suffer from potential co-variation issues, see discussion for details). For the seagrasses, we extracted data on (1) abundance (shoot density; some studies did not report this and these studies were excluded from the analysis), (2) maximum leaf size (small-sized species = *Halodule wrigthii, Halophila ovalis*; medium-sized species = *Cymodocea nodosa*, *Zostera noltii*; large-sized species = *Amphibolis* sp., *Enhalus acoroides*, *Thalassia hemprichii*, *T. testudinum*, *Z. marina*) [2], and (3) taxonomic identity (genus). Supplementary tests of modifying effects of habitat/methodological conditions are shown in Appendix S2 and Figure S1.

We extracted corresponding means, measures of dispersion (SD, SE, or CL) and replication levels for all reported seagrass responses reported on the individual or population level (e.g., leaf length, survival, growth, reproduction, density, biomass) from all experiments. For repeated measures designs, we only included the last reported data point, a standard practice in ecological meta-analysis [23]. Thus, we extracted all seagrass responses where plots without the seaweeds were compared to plots with seaweeds, including multiple seaweed abundance levels, seagrass responses, and orthogonal and nested designs.

Hedge's effect size *d*, corrected for small sample sizes, was used to calculate standardized impacts [27]. This metric allows, in contrast to the response-ratio metric, the usage of reported zero-value responses [23], [24]. ‘Treatments’ were defined as plots with seaweeds, and ‘controls’ as plots without seaweeds; *d* values are therefore negative if the seaweed causes a reduction in seagrass responses. First, we calculated individual values of *d* ( = *d*_individual_) for each reported response within any given experiment. For example, an experiment could report impact on both seagrass biomass and growth ( = 2 *d*_individual_). These within-experimental *d*-values are strongly auto-correlated [10]. Second, we calculated average effect sizes for each experiment (*d*_experiment_), using equal weight for all the *d*_individual_ that were reported per experiment (i.e., we assumed biomass and growth were equally important in our example above). Cumulative meta-analyses were conducted on these ‘independent’ effect sizes. Continuous (e.g., experimental run-time) attributes were analyzed with meta-analytical linear regression (*d*_experiment_ against predictor values). Categorical (e.g., attachment type) attributes were analyzed with categorical analysis, by averaging multiple *d*_experiment_ into a single *d*_cummulative_ for each treatment [27]. We used random-effect models because these models assume that summary statistics have both sampling error and a true random component of variation in effect sizes between studies. We report results calculated as 95% bias corrected confidence limits (from 999 iterations), but results were generally similar for both standard and bootstrap calculated confidence limits (see Appendix S3). For the extra categorical analysis on seaweed abundance effects, we tested if the difference between paired *d*_experiment_ values (Δ*d*_experiment_ = *d*_high-abundance_−*d*_low abundance_ within a specific experiment) was significantly different from zero [28], were Δ*d* is negative if abundant seaweed have larger negative effect than sparse seaweeds. If a test was significant, the individual treatments were compared graphically; i.e., treatments were interpreted conservatively to be significantly different from zero or each other, if confidence limits did not overlap zero or each other. All tests were conducted both as weighted and un-weighted analyses; experiments with low replication and/or high data variability were considered less important in the former case, whereas all experiments were considered of equal importance in the latter. Results were generally similar between analyses and we here present the weighted case (the un-weighted results are shown in Appendix S3). Analyses of publication bias are presented in Appendix S4 and Figure S2. All meta-analyses were conducted in MetaWin 2.0 [27].

We had *a priori* simple expectations about the direction and relative magnitude of effect sizes between treatments for several of the impact attributes (beyond the notion that seaweed have negative impact on seagrass performance). We expected larger negative effect sizes when there was more of the seaweed (in space or time) and/or less of the impacted seagrass (e.g., in density or size). We had no similar expectation about differences in effect sizes between different attachment types, morphologies or taxonomies (see Table 1 for details).

**Table 1:** Attributes of seaweeds and seagrasses that may influence seaweed impact on seagrass.

Attribute^1^	Seaweedhypotheses	SeaweedResults	Seagrasshypotheses	Seagrassresults
Abundance (per area): High *vs.* Low	H>L	H>L (**CL≠0**)^2^	H<L^3^	H = L (p = 0.254)
Size (per individual): Large *vs.* Small	L>S	NT	L<S	L<S (**p = 0.035**)
Extent (plot size): Large *vs.* Small	L>S	S>L (**p<0.01**)	L<S	NT
Duration (run time): Long *vs.* Short	L>S	S>L (**p<0.01**)	L<S	NT
Origin^4^: Native *vs.* Invasive	N<I	N>I (**p = 0.02**)	I<N	NT
‘Condition’: Healthy *vs.* Decomposing	D>H^5^	NT	H<D	NT
Clonal/Modular: Integrated *vs.* Solitary	I>S	NT	I<S^6^	NT
Attachment	?	Dri = Epi > Roo (**p = 0.049**)^7^	?	NT^8^
Morphology	?	She≥Coa≥Fil>Coe^9^ (**p = 0.02**)	?	NT
Taxonomy (genus)	?	Ulv≥Gra≥Lau≥Cau^10^ (**p<0.01**)	?	Amp<Cym = Tha≤Zos≤Had<Hap^11^ (**p<0.01**)

**Figure 1:**
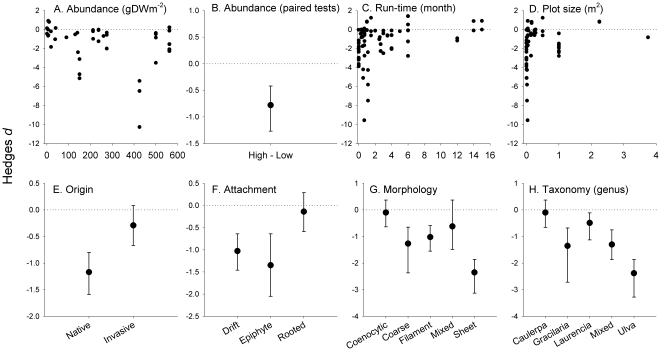
Effects of seaweed attributes on seagrass performance. Hedges *d* represent *d*_experiment_ for continuous data and *d*_cumulative_ ±95% CL for categorical data. Data were extracted from up to 59 experiments. Fig. B is based on 17 experiments that tested explicitly for abundance effects. Effects are here reported as Δ*d* = *d*_high_−*d*_low_; if Δ*d* is negative then high abundance cause larger negative effect than low abundance. Fig. G: coenocytic = single celled seaweed with modular growth of interconnected fronds. For meta-analytical test results and sample sizes, see Appendix S3.

**Figure 2:**
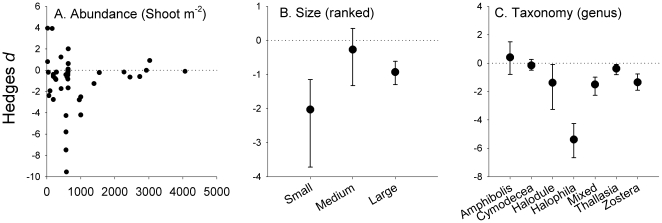
Modifying effects of seagrass attributes on seaweed impacts. Hedges *d* represent *d*_experiment_ for continuous and *d*_cumulative_ ±95% CL for categorical data. Data were extracted from up to 59 experiments. For meta-analytical test results and sample sizes, see Appendix S3.

We had *a priori* expectations about the direction of impact for the first seven attributes (above the dotted line). These directional hypotheses are based on simple rules; we expect a large impact when there is (a) *more* of a stressor (the seaweed) in either space or time, or (b) *less* of the impacted organism (the seagrass). Summary of tests-results are shown in the table (significant values in bold, see also Fig. 1, 2 and Appendix S3; NT = not tested because data were inadequate).

1 Impact of seaweeds on seagrasses may also be modified by habitat attributes, including the resource levels (e.g., nutrients, light, O_2_, space), abiotic conditions (e.g., temperature, salinity, desiccation, sedimentation, substrate conditions, day-length) and resident animals living in and around the seagrass habitat [21].

2 The categorical test based on experiments that explicitly tested for abundance effect was significant, but the correlation conducted across all experiments was not significant.

3 We assume that abundant seagrasses have more resources to withstand stress. Alternatively, abundant seagrass may suffer from intra-specific competition resulting in abundant seagrass being more susceptible to stress (i.e. the opposite expectation may be equally valid).

4 We assume that invaders have superior impact (seaweeds) and resistance (seagrass), e.g., as novel weapons [44].

5 Poor ‘condition/health’ of the seaweed results in decomposition and production of anoxia, sulphide and ammonia. Unattached mats often decompose when lower layers are shaded by higher layers.

6 For seagrasses, integration is a continuous attribute that encompasses below ground storage products and ability to translocate products between ramets.

7 Dri = Drift/unattached, Epi = epiphytic to seagrass leaves, Roo = rooted in sediment with rhizoids and rhizomes.

8 A few seagrasses can attach to rocks, but no studies have quantified seaweed impacts on attached seagrass.

9 Adapted from Littler and Littler (1980); She = sheets, Coa = Coarsely branches, Fil = filaments, Coe = coenocytic.

10 Ulv = *Ulva*, Gra = *Gracilaria*, Lau = *Laurencia*, Cau = *Caulerpa.*

11 Amp = *Amphibolis*, Tha = *Thalassia*, Cym = *Cymodocea*, Zos = *Zostera*, Had = *Halodule*, Hap = *Halophila*.

## Results

We calculated 381 *d*_individual_ from the 59 experiments published in 22 studies; most studies were conducted in the Atlantic Ocean; 9 in the Northeast (including 3 in the Mediterranean Sea) and 7 in the Northwest (including 1 in Gulf of Mexico and 1 in the Caribbean Sea). By contrast, only three studies were conducted in the Pacific Ocean – two in the Northeast and one in the Southwest. Similarly, three studies were conducted in the Indian Ocean - two in the East and one in the West (Appendix S1). Of the 22 studies, 13 were conducted in relatively warm waters (including Mediterranean studies) and 9 in relatively cool waters (including Portuguese Atlantic studies). The cumulative effect size calculated from all 59 average *d*_experiment_ was −0.96 (95% bias corrected CL = −1.28 to −0.65, Q_total_ = 83.34, p = 0.01) documenting that, overall and across all studies, species and abiotic conditions, seaweeds have negative impact on seagrasses.

The regression analysis on continuous seaweed abundances was not significant (p_slope_ = 0.98, Fig. 1A). However, the categorical analysis on paired effects showed that, in those experiments that explicitly tested for abundance effects, impact was significantly more negative at high, compared to low, seaweed abundances (confidence limits of Δ*d* did not overlap zero, Fig. 1B). We found positive effects of both increasing experimental duration (p_slope_<0.01, Fig. 1C) and plot size (p_slope_<0.01, Fig. 1D). There was a significant effect of seaweed origin (p = 0.02) with native seaweeds having larger negative effects than invasive seaweeds (Fig. 1E). We also found significant effects of seaweed attachment types (p = 0.049), where drift algae and epiphytes caused more negative effects that rooted algae (Fig. 1F). Seaweed morphology also influenced impact (p = 0.02); sheet-forming, filamentous and coarsely-branched seaweeds had larger negative effect than the coenocytic/clonal morphologies (Fig. 1G, coenocytic seaweeds were not different from zero). Finally, we also found significant effects of seaweed taxonomy (p<0.0l), with large negative effects of *Ulva* species, intermediate negative effects of *Gracilaria*, and small, but still significant, negative effects of *Laurencia*. By contrast, effects of *Caulerpa* were not significantly different from zero (Fig. 1H).

For the seagrass attributes, we found no effects of seagrass abundance (p_slope_ = 0.24, Fig. 2A). However, the size of seagrass species influenced impact (p = 0.035) with small species being significantly more negatively affected than large species (Fig. 2B, note that the large error bars of intermediate sizes species overlapped zero). Seagrass taxonomy also influenced impact (p<0.01) with large negative effects observed on *Halophila* species, intermediate negative effects on *Halodule* and *Zostera* species and small, but still significant, negative effect on *Thalassia* species (Fig. 2C). There were no significant effects on *Cymodocea* or *Amphibolis* species (confidence limits overlapping zero).

## Discussion

Seaweeds have been argued to be a significant cause of seagrass declines around the world [7], [8], [9], [18]. However, experimental evidence for impacts on seagrasses only exists from 22 published studies. We reviewed these studies using standardized and quantitative methods (meta-analysis). These analyses confirmed that seaweeds have negative impacts on seagrasses at the scale of patches (here<5 m^2^). More specifically, we documented that the abundance of the seaweeds and their attachment type, and the size of the seagrass, are particular important attributes that determine the magnitude of negative impact. Our quantitative review of published studies, also allowed us, indirectly, to list significant research gaps. Below we discuss our findings.

### Hypotheses about directionality of effect sizes

Only two of our directional hypotheses (Table 1) were supported; impacts were large when seaweeds were abundant and when seagrasses were small (short leaves). Several studies have tested for effects of abundance [10], [26], [29], and this allowed us to conduct an un-confounded impact analysis (comparing effects of abundant *vs.* sparse treatments, Fig. 1B). While intuitively simple, this result provides rigorous quantitative support to the qualitative notion that seaweed abundance, no matter the species, abiotic conditions or resource levels, is a critical parameter to consider to understand impacts on seagrasses.

**Figure 3:**
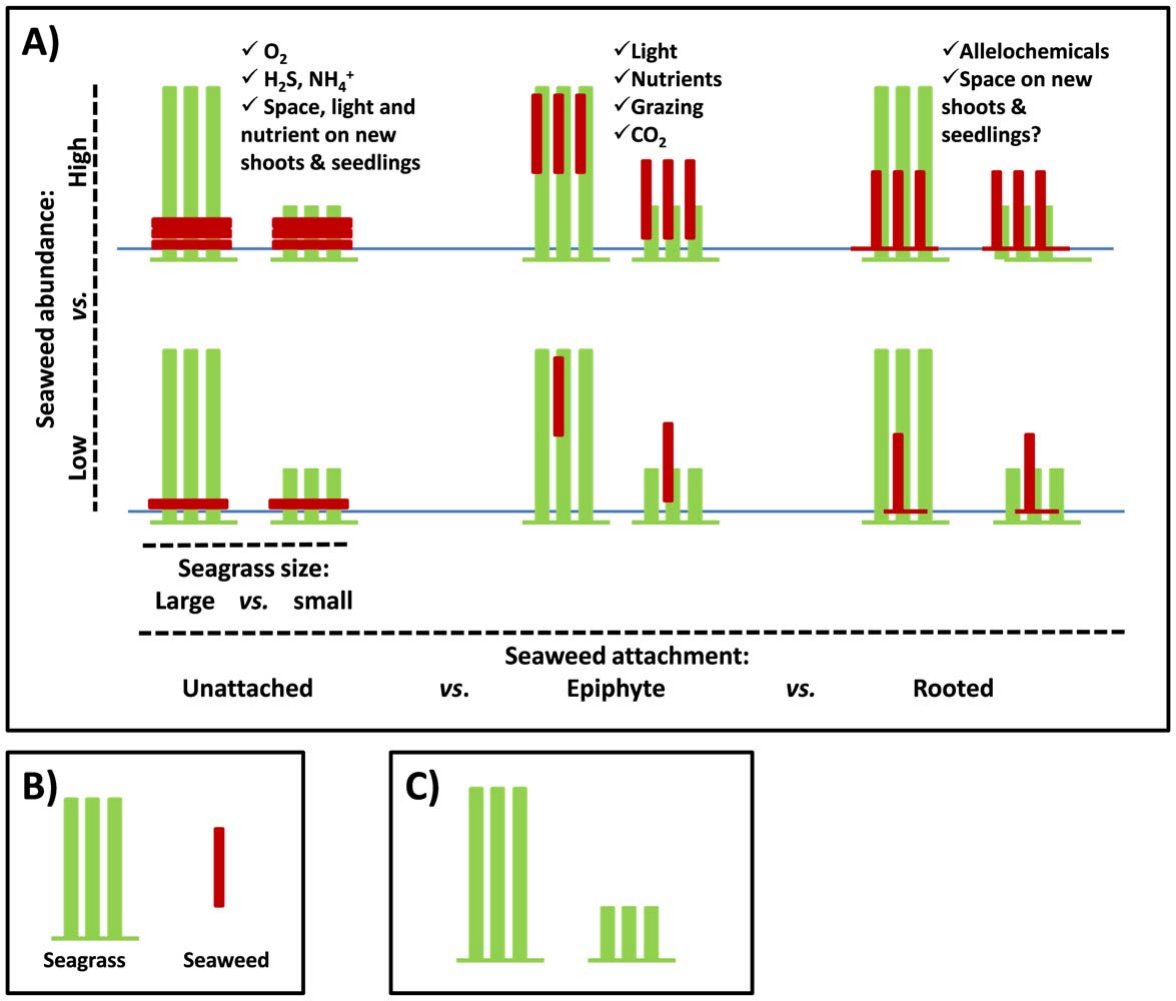
Seaweed impacts on seagrasses can be partially predicted from basic impact attributes. *Plot 3A: Key meta-analytical results schematized* (Fig. 1–2, Table 1). Impact depends on seaweed abundance (low *vs.* high, cf. y-axis), seaweed attachment (unattached *vs.* epiphytic *vs.* rooted, cf. long x-axis) and seagrass size (large *vs.* small, cf. short x-axis). The impact mechanisms associated with seaweed abundance and seagrass size are simple; the more of the stressor (seaweed) and less of the impacted organism (seagrass) the larger the impact. The mechanisms that cause different effects between attachment types are less obvious; we suggest that oxygen and light reduction and sulphide production cause large negative impact of unattached and epiphytic seaweeds, whereas allelochemicals cause smaller impacts of rooted seaweeds (listed in bullets). Our analysis addressed impact attributes in isolation. Future tests should use factorial designs to identify interactions between attributes. *Plot 3B: Figure legend*. Standardized seagrass = three green leaves connected with rhizomes; leaves can be large or small. Standardized seaweed = brown frond; can be sparse or abundant (1 *vs.* 3 fronds), positioned vertical (attached *vs.* rooted) or horizontal (unattached), and with (rooted) or without (unattached, attached) inter-connecting rhizome. *Plot 3C: Non-impacted controls.* The impact treatments shown in plot 3A should always be compared to non-impacted seagrass controls, here to ‘large and small seagrass without seaweed stress’.

In contrast to the seaweed abundance test, no experimental studies have tested if seagrass size *per se* modifies how seaweeds impact seagrasses. It is therefore possible that the large impact observed on small seagrasses co-vary with other seagrass-attributes, such as their longevity, clonal integrity, shoot density or taxonomic identity (i.e., small species are generally ephemeral, have low clonal integration, high shoot density and belong to the genera *Halophila* and *Halodule*). Future studies should conduct un-confounded experiments on how seagrass size (leaf length) modifies seaweed impact; for example, by comparing seaweed impact on small *vs.* large leaves and on seedlings *vs.* established leaves of the same species or ecotype (e.g. as in [30] - although this test is confounded by ontogeny), or by comparing impacts on small and large seagrass species using a random subset of species or ecotypes as a nested factor within each size class. Despite co-variation issues, we believe that seaweed abundance and seagrass size reflect fundamental first-order attributes of seaweed-seagrass interactions that affect the impact (Fig. 3). For example, low *Gracilaria* density had virtually no effects on the relatively large *Zostera marina* seagrass [13], whereas high *Gracilaria* density had detrimental effects on the smaller *Halophila ovalis* seagrass [10].

We found that duration and spatial extent of seaweed stress (by proxy of experimental run-time and plot size, respectively) correlated positively with hedges *d* effect sizes, not negatively as expected (Table 1). Except from a single experiment that explicitly quantified impact over different independent time intervals [31; this study did not find effect of duration of stress], our results were evaluated from studies designed to test for other attributes, such as seaweed abundance or modifying effects of nutrient or temperature [10], [13], [32]. Positive slopes may therefore be caused by co-varying attributes. For example, it may be difficult to maintain seaweed densities in long experiments, e.g. due to increased likelihood of encountering storms [33], [34], [35], [36], [37], and seaweed sizes may decrease over time due to phenological changes [38]. Experiments conducted on short time-scales, on the other hand, are more often conducted in the laboratory, where seaweed densities are easier to maintain and where impacts are measured on seagrass planting units with limited storage-reserves to resist stress [10], [26]. Similar co-variation is likely to occur with plot-size; small-plot experiments are more often conducted in the laboratory and measured on short time-scales on physiological performances (e.g., photosynthesis of leaves) [39], compared to large-plot experiments that are conducted in the field with intact clonal integration and impact measured on whole-plot performances (e.g., total above ground biomass) [37]. In short, the reported positive slope is most likely a combined result of logistical problems (it is difficult to maintain high densities over long time and in large plots in field experiments) and co-variation issues (small and large plot experiments are relatively more often conducted in the laboratory and field, respectively, see also Appendix S2).

Non-native seaweeds can arrive to seagrass beds through different transport vectors. Most seaweed introductions stems from unintentional arrivals to the new regions attached to imported oysters, on ship hulls, as accidental releases from aquaria, or via canals, like the Suez canal [12]. However, a few introductions are intentional, e.g., *Gracilaria* and *Eucheuma* have been introduced for aquaculture (used to produce phycocolloids) [40]. These farmed seaweeds can have negative impact on the seagrass [40], [41], but we are not aware of studies that link transport vectors and impact on seagrass. This could be an important future research topic; for example, intentional introduced seaweeds may have strongest impact on seagrass if they are ‘nursed’ by humans. We found, unexpectedly, that invasive seaweeds had lower negative effects than native species. Importantly, co-evolution between seaweeds and seagrasses may be weaker than anticipated, i.e., there may not be any reason to expect why non-native species should have stronger impact than native species. Instead, co-varying impact attributes can cause the reversed pattern as observed, because many experiments with invasive seaweeds have been conducted using rooted *Caulerpa* species (Fig. 1F, G, I). Thus, it may be that the low invasion impact reported here reflects the relatively small effects sizes observed for *Caulerpa*, rather than where seaweeds originate from (for more detail on co-variation issues, see discussion on attachment type and taxonomy, below). So far, no experiments have tested if invasive seaweeds *per se* have larger impacts than native seaweeds (e.g., by testing if a particular seaweed have different effects in its native or introduced region), even though this test is repeatedly called for in the invasion literature [21], [42]. Alternatively, it has been suggested that invasive species can be more susceptible to native enemies and local abiotic stressors, compared to native species [43], [44], and this could perhaps translate into smaller impacts reported from manipulative experiments using non-native seaweeds. It is of course also possible that some of the seaweeds that have been classified as native could, in fact, be invasive (e.g. *Gracilariopsis*, *Enteromorpha*), as it has been documented through biogeographic and molecular analyses of other seaweed blooms [45]. Finally, we did not find support for our expectation that high seagrass densities resulted in higher resistance to seaweed stress (i.e., a lowered impact). Only a single study has specifically tested this, finding a weak modifying effect [46]. Again, co-variation between attributes may influence results; species with high densities are typically small species (*Halophila*, *Halodule*) that are susceptible to seaweed impact (Fig. 3B).

### Hypotheses about non-directional effect sizes

All tests without *a priori* directional expectations (Table 1) were significant; the attachment type, morphology and taxonomy of the seaweed and the taxonomy of the seagrass all predict impacts. Again, these attributes co-vary. For example, rooted seaweeds (Fig. 1F) are coenocytic species (Fig. 1G) that belong to the genera *Caulerpa* and *Halimeda* (Fig. 1I). Similarly, unattached seaweeds are generally sheet-forming or coarsely branched algae, belonging to the genera *Ulva* and *Gracilaria*, respectively. Epiphytic algae are typically represented by a mixture of filamentous species and very few species-specific impact data exists (Appendix S1). These co-variation issues are difficult to disentangle because of the lack of independence among the studied attributes, i.e., they depend inherently on genetic traits in contrast to abundance, density and size attributes that, at least in theory, can be similar between different species.

Impacts by sheet-forming and coarsely-branched unattached algae were more negative than coenocytic rooted seaweeds (Fig. 1G). Differences in allelochemical interactions seems an unlikely cause because species belonging to *Caulerpa* and *Halimeda* (with reported low impact) often contain high levels of toxins [47], [48]. Instead, we suggest that the horizontal position at the sediment surface of unattached seaweeds shade small seagrasses and seedlings, and - more importantly - reduce gas exchange compared to the upright position of rooted seaweeds. Unattached horizontal seaweeds thereby create short and strong vertical gradients in light, oxygen and (toxic) ammonia and sulphide [26], [49], [50], [51], [52], resulting in adverse conditions for the sensitive seagrass meristem positioned basally near the sediment surface [53], [54]. It is also possible that impacts of unattached seaweeds have been tested with higher biomass than rooted seaweeds, a confounding effect that is difficult to quantify because the abundance of the rooted seaweeds typically is reported as frond densities instead of biomass [17], [32], [55], [56]. Perhaps genetic constraints pose physical limitations to the length and density of rooted fronds. Genetic limitations ultimately define how efficiently rooted seaweeds can use up resources; rooted seaweeds typically grow fronds<30 cm long and many have open space between interconnected frond (Fig. 3). There are less constraints to the size and compactness for unattached seaweeds. Unattached seaweeds may continue to accumulate (e.g., transported by currents) into thicker and denser mats, creating high biomass per area, resulting in efficient space occupation, light interception, nutrient filtering and, most importantly, high production of anoxia and sulphide levels [26], [49], [50]. It is vital that future tests compare impacts between different attachment types, morphologies and taxonomic identities using similar abundances and experimental conditions (Fig. 3).

### Research gaps

In our review so far, we have outlined some important research gaps; for example, future experiments should test for effects of seagrass size (within an ecotype), for duration and plot size (within a single experiment) and attachment types and morphology of the seaweeds, explicitly aiming to reduce co-varying/confounding issues. These examples are included in a more comprehensive list of studies that are needed to be able to predict precisely how seaweed impacts seagrass (Appendix S5). Rather than addressing each gap in detail, we simply highlight that targeting these gaps does not necessarily require sophisticated equipment, or highly advanced methodologies, but rather reflects an urgent need for labour-intensive ‘simple-but-hard-work’. For example, we only found a few studies which manipulated seagrass epiphytes [31], [46], [57] (using simple but efficient hand-picking), even though the problem of seagrasses being covered by epiphytes has been known around the world for decades [57], [58]. Thus, most of the proposed research gaps can be addressed with relatively limited means. In short, we argue here that not a single impact attribute (research gaps 1–5), their interactions (gap 6) or the broader ecological context of seaweed-seagrass interactions (gaps 7–12) have yet been studied in adequate detail to provide the necessary background information that allows managers and scientists to model and predict seaweed impacts on seagrasses at the local patch scale. However, we also believe that rapid progress is possible if the necessary logistic and labour-intensive resources are allocated.

### Conclusion

We detected large variability of impacts of seaweeds on seagrasses in the reviewed experiments, and many types of co-variation between which makes it difficult to pinpoint what attributes drive impacts. Hence, only the most robust and general attributes could be confirmed to influence impact across the reviewed studies; seaweed abundance and attachment type (which co-vary strongly with seaweed morphology and taxonomy) and seagrass size (which co-vary strongly with seagrass taxonomy) modify the magnitude of stress impact. These attributes, therefore, provide baseline models for how seaweeds impact seagrasses (Table 1, Fig. 1, 2, 3). We also suggest that impact attributes should be tested in much more detail and with factorial approaches to develop more realistic impact models and to prioritize and evaluate their relative importance (Appendix S5). Finally, we hope that this review will stimulate progress in seaweed-impact ecology, ultimately providing managers and scientists with improved tools to conserve rapidly deteriorating seagrass beds around the world.

## Supporting Information

Figure S1 **Modifying effects of seagrass habitat attributes on seaweed impact on seagrass performance.** Data extracted from up to 59 experiments. For meta-analytical test results and sample sizes, see online S3.
(TIF)

Figure S2 **Funnel plot of average effect sizes (Hedges** ***d*** **) from 59 experiments.**
(TIF)

Checklist S1 **Prisma 2009 checklist for meta-analytical reviews.**
(DOC)

Diagram S1 **Prisma 2009 flow diagram for meta-analytical reviews.**
(DOC)

Appendix S1 **Reviewed experimental studies used to extract effects of seaweeds on seagrasses.**
(DOC)

Appendix S2 **Modifying effects of habitat and methodology on seaweed impact on seagrasses.**
(DOC)

Appendix S3 **Meta-analytical test results and sample sizes.**
(DOC)

Appendix S4 **Publication bias.**
(DOC)

Appendix S5 **List of research gaps in seaweed-seagrass interaction studies.**
(DOC)
