# Supplementary material for: A Meta-Analysis of Seaweed Impacts on Seagrasses: Generalities and Knowledge Gaps
Source: PLoS One. 2012 Jan 10;7(1):e28595. doi: 10.1371/journal.pone.0028595 (PMC3254607; doi:10.1371/journal.pone.0028595)
Supplement: Diagram S1 — Prisma 2009 flow diagram for meta-analytical reviews. (DOC) [file pone.0028595.s004.doc]

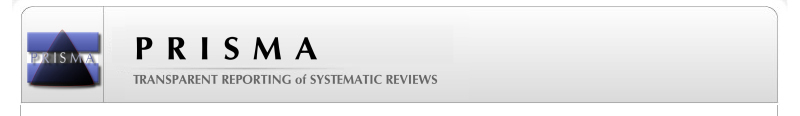
**PRISMA 2009 Flow Diagram**

Note: We have conducted 4 of the most recent studies of the types of studies we searched for, and therefore had exhaustive hands-on insights into the available limited literature.

**Screening**

**Included**

**Eligibility**

**Identification**

Records excluded

[data anecdotal or not about seaweed impact (as stressor) or seagrass (as focal organism]
(n = 350)

Full-text articles excluded, with reasons

[data anecdotal, mensurative (no control over seaweed abundances), impact not on seagrass, or impact on sub-individual levels such as cellular fluorescence]
(n = 99 )

Records identified through database searching
(n = 420 )

Additional records identified through other sources
(n = 51 )

Records after duplicates removed
(n = 471)

Records screened (from abstracts) (n = 471 )

Full-text articles assessed for eligibility
(n = 121)

Studies included in qualitative synthesis
(n = 22)

Studies included in quantitative synthesis (meta-analysis)
(n = 22)
