# Supplementary material for: A Meta-Analysis of Seaweed Impacts on Seagrasses: Generalities and Knowledge Gaps
Source: PLoS One. 2012 Jan 10;7(1):e28595. doi: 10.1371/journal.pone.0028595 (PMC3254607; doi:10.1371/journal.pone.0028595)
Supplement: Appendix S1 — Reviewed experimental studies used to extract effects of seaweeds on seagrasses. (DOC) [file pone.0028595.s005.doc]

# Appendix S1: Reviewed experimental studies used to extract effects of seaweeds on seagrasses

Seaweed-column: *taxonomy* = Green, Red, Mixed/Multiple; *attachment* = Rooted, Drift, Epiphyte, Mixed/Multiple; *morphology* = Clonal, Coarsely-branched, Sheet-form, Filamentous, Encrusting-calcareous, Articulated-calcareous. Species names that are underlined are non-natives. Seagrass-column: Number in brackets = ranked ‘size’. Experimental-design column: ‘×’ = orthogonal test-factor. Data-column: show the figures and tables from where data was extracted for the meta-analysis.

| **Study, region** | **Seaweed**  **(taxonomy, attachment, morphology)** | **Seagrass** | **Experimental design** | **Data** |
| --- | --- | --- | --- | --- |
| [1]: Italy, Mediterranean sea | *Caulerpa taxifolia* (G,R,Clo) | *Cymodocea nodosa* (2) | 2 seaweed treatments × 2 neighbours [=competition] × 2 nutrients × 8 sites | Fig. 3 |
| [2]: Italy, Mediterranean sea | *Caulerpa racemosa* (G,R,Clo) | *Zostera noltii* (2)*, Cymodocea nodosa* (2) | 2 seaweed treatments × 2 sites | Fig. 1, 2, 3 |
| [3]: Italy, Mediterranean sea | *Caulerpa taxifolia* (G,R,Clo) | *Cymodocea nodosa* (2) | 2 seaweed treatments × 2 neighbours [=competition] × 2 nutrients × 8 sites | Fig. 2 |
| [4]: USA, NW Atlantic | *Caulerpa prolifera* (G,R,Clo) | *Halodule wrigthii* (1) | 2 seaweed treatments × 2 neighbours [=competition] × 2 depth | Fig. 3, 2 |
| [5]: Tanzania, West Indian Ocean | *Eucheuma dentticulatum* (R,D,Coa) | *Enhalus acoroides* (3)*, Thalassia hemprichii* (3) | 3 seaweed treatments [including cage-artefact] | Fig. 1, 2, 3, 4 |
| [6]: USA, Caribbean | *Laurencia poiteaui* (R,D,Coa) | *Thalassia testudinum* (3) (w/*Halodule wrightii, Syringodium filiforme*) | 2 seaweed treatments | Fig. 1-5 |
| [7]: USA, NE Pacific | *Ulvaria obscura* (G,D,S) | *Zostera marina* (3) | 3 seaweed treatments [impact *vs.* two non-impacted controls] | Fig. 1, Table 1 |
| [8]: USA, NW Atlantic | Mixed (*Laurencia, Dictyota*) (M,D,M) | *Thalassia testudinum* (3) | 3 seaweed treatments [including cage-artefact] × 3 grazers | Fig. 1 |
| [9]: Australia, SW Pacific | *Enteromorpha intestinalis* | Mixed (*Halophila ovalis* 60%, *Zostera capricorni* (20%), *Ruppia megacarpa* (20%) | 3 seaweed treatments [including cage-artefacts] × 2 sites | Fig. 3, 4, Tabl 1 |
| [10]: Denmark, NE Atlantic | Mixed (*Ceramium, Chaetomorpha*) (M,D,Fil) | *Zostera marina* (3) | 3 seaweed abundances | Fig. 3, 4, Table 2 |
| [11]: USA, NW Atlantic | *Laurencia* (R,D,Coa) | *Thalassia testudinum* (3) | 2 seaweed treatments × 6 times [=incubation length = experiment 1] | Fig. 3 |
| [11]: USA, NW Atlantic | *Laurencia* (R,D,Coa) | *Thalassia testudinum* (3) | 2 seaweed treatments × 2 experiments [=time, methods = experiment 2) | txt p87 |
| [11]: USA, NW Atlantic | Epiphytes – encrusting (M,E,Enc-cal) | *Thalassia testudinum* (3) | 2 seaweed treatments [=experiment 3) | txt p87 |
| [11]: USA, NW Atlantic | *Laurencia vs.* Encrusting epiphytes) | *Thalassia testudinum* (3) | 3 seaweed attachment types [=epiphytes *vs.* drift = experiment 4] | txt p87 |
| [12]: USA, Gulf of Mexico | Mixed drift (M,D,M) | *Thalassia testudinum* (3) | 2 seaweed treatments [also test for effect of abiotic light reduction and follow recovery after drift removal] | Fig. 1, 3, 4, 5 |
| [13]: USA, NE Pacific | *Gracilariopsis* sp. (R,D,Coa) | *Zostera marina* (3) | 4 seaweed abundance [including cage-artefact] | Fig. 4, 5 |
| [14]: USA, NW Atlantic | Mixed (*Gracilaria tikvahia, Cladophora vagabunda*) (M,D,M) | *Zostera marina* (3) | 5-6 seaweed abundance [including cage-artefact] × 2 sites | Fig. 2, 5, 6 |
| [15]: Denmark, NE Atlantic | *Gracilaria vermiculophylla* (R,D,Coa) | *Zostera marina* (3) | 3 seaweed abundance × 4 temperature | Fig. 1-3 |
| [16]: Portugal, NE Atlantic | *Ulva rigida* (G,D,She) | *Zostera noltii* (2) | 4 seaweed abundance | Fig. 1 |
| [17]: Portugal, NE Atlantic | *Ulva rigida* (G,D,She) | *Zostera noltii* (2) | 4 seaweed abundance × 3 experiments [=lab, field1, field2] | Fig. 1-3 |
| [18]: Australia, East Indian Ocean | Epiphytes (mixed) (M,E,fil) | *Amphibolis* sp. (3) | 2 seaweed treatments × 2 seagraass abundance | Fig. 1 |
| [19]: USA, NW Atlantic. | *Halimeda incrasata* (G,R,Ere-cal) | *Thalassia testudinum* (3) | 3 seaweed abundance × 2 neighbours [=competition] | Fig. 3-5 |
| [20]: Denmark, NE Atlantic | Epiphytes (diatoms) (M,E,fil) | *Zostera marina* (3) | 2 seaweed treatments × 6 HCO3 | Fig. 1 |
| [20]: Denmark, NE Atlantic | Epiphytes (diatoms) (M,E,fil) | *Zostera marina* (3) | 2 seaweed treatments × 7 light | Fig. 4 |
| [21]: Australia, East Indian Ocean | *Gracilaria comosa* (R,D,Coa) | *Halophila ovalis* (1) | 3 seaweed abundance × 3 temperature | Fig. 1-3 |
| [22]. Denmark, NE Atlantic | *Gracilaria vermiculophylla* (R,D,Coa) | *Zostera marina* (3) | 3 seaweed abundance × 3 experiments [=temperature] | Fig. 5-6 |

# References

1. Ceccherelli G, Cinelli F (1997) Short-term effects of nutrient enrichment of the sediment and interactions between the seagrass *Cymodocea nodosa* and the introduced green alga *Caulerpa taxifolia* in a Mediterranean bay. Journal of Experimental Marine Biology and Ecology 217: 165-177.

2. Ceccherelli G, Campo D (2002) Different effects of *Caulerpa racemosa* on two co-occuring seagrasses in the Mediterranean. Botanica Marina 45: 71-76.

3. Ceccherelli G, Sechi N (2002) Nutrient availability in the sediment and the reciprocal effects between the native seagrass *Cymodocea nodosa* and the introduced green alga *Caulerpa taxifolia* in a Mediterranean bay. Hydrobiologia 474: 57-66.

4. Taplin KA, Irlandi EA, Raves R (2005) Interference between the macroalga *Caulerpa prolifera* and the seagrass *Halodule wrightii*. Aquatic Botany 83: 175-186.

5. Ekloef JS, Henriksson R, Kautsky N (2006) Effects of tropical open-water seaweed farming on seagrass ecosystem structure and function. Marine Ecology Progress Series 325: 73-84.

6. Holmquist JG (1997) Disturbance and gap formation in a marine benthic mosaic - influence of shifting macroalgal patches on seagrass structure and mobile invertebrates. Marine Ecology Progress Series 158: 121-130.

7. Nelson TA, Lee A (2001) A manipulative experiment demonstrates that blooms of the macroalga *Ulvaria obscura* can reduce eelgrass shoot density. Aquatic Botany 71: 149-154.

8. Marcia S (2000) The effects of sea urchin grazing and drift algal blooms on a subtropical seagrass bed community. Journal of Experimental Marine Biology and Ecology 246: 53-67.

9. Cummins SP, Roberts DE, Zimmerman KD (2004) Effects of the green macroalgae *Enteromorpha intestinalis* on macrobenthic and seagrass assemblages in a shallow coastal estuary. Marine Ecology Progress Series 266: 77-87.

10. Holmer M, Nielsen RM (2007) Effects of filamentous algal mats on sulfide invasion in eelgrass (*Zostera marina*). Journal of Experimental Marine Biology and Ecology 353: 245-252.

11. Irlandi EA, Orlando BA, Biber PD (2004) Drift algae-epiphyte-seagrass interactions in a subtropical *Thalassia testudinum* meadow. marine Ecology Progress Series 279: 81-91.

12. Lamote M, Dunton KH (2006) Effects of drift macroalgae and light attenuation on chlorophyll fluorescence and sediment sulfides in the seagrass *Thalassia testudinum*. Journal of Experimental Marine Biology and Ecology 334: 174-186.

13. Huntington B, Boyer KE (2008) Effects of red macroalgal (*Gracilariopsis* sp.) abundance on eelgrass *Zostera marina* in Tomales Bay, California, USA. Marine Ecology Progress Series 367: 133-142.

14. Hauxwell J, Cebrian J, Furlong C, Valiela I (2001) Macroalgal canopies contribute to eelgrass (*Zostera marina*) decline in temperate estuarine ecosystems. Ecology: 1007-1022.

15. Martinez-Luscher J, Holmer M (2010) Potential effects of the invasive species *Gracilaria vermiculophylla* on *Zostera marina* metabolism and survival. Marine Environmental Research 69: 345-349.

16. Brun FG, Hernandez I, Vergara JJ, Perez-Llorens JL (2003) Growth, carbon allocation and proteolytic activity in the seagrass *Zostera noltii* shaded by *Ulva* canopies. Functional Plant Biology 30: 551-560.

17. Brun FG, Vergara JJ, Navarro G, harnandez I, Perez-Llorens JL (2003) Effect of shading by *Ulva rigida* canopies on growth and carbon balance of the seagrass *Zostera noltii*. Marine Ecology Progress Series 265: 85-96.

18. Edgar GJ, Robertson AI (1992) The influence of seagrass structure on the distribution and abundance of mobile epifauna: pattern and processes in a Western Australian *Amphibolis* bed. Journal of Experimental Marine Biology and Ecology 160: 13-31.

19. Davis BC, Fourqurean JW (2001) Competition between the tropical alga, *Halimeda incrassata*, and the seagrass, *Thalassia testudinum*. Aquatic Botany 71: 217-232.

20. Sand-Jensen K (1977) Effect of epiphytes on eelgrass photosynthesis. Aquatic Botany 3: 55-63.

21. Holmer M, Wirachwong P, Thomsen MS (2011) Negative effects of stress-resistant drift algae and high temperature on a small ephemeral seagrass species. Marine Biology 158: 297-309.

22. Höffle H, Thomsen MS, Holmer M (2011) High mortality of *Zostera marina* under high temperature regimes but minor effects of the invasive macroalgae *Gracilaria vermiculophylla*. Estuarine, Coastal and Shelf Science 92: 35-46.
