# Supplementary material for: A Meta-Analysis of Seaweed Impacts on Seagrasses: Generalities and Knowledge Gaps
Source: PLoS One. 2012 Jan 10;7(1):e28595. doi: 10.1371/journal.pone.0028595 (PMC3254607; doi:10.1371/journal.pone.0028595)
Supplement: Appendix S2 — Modifying effects of habitat and methodology on seaweed impact on seagrasses. (DOC) [file pone.0028595.s006.doc]

# Appendix S2. Modifying effects of habitat and methodology on seaweed impact on seagrasses

*Introduction.* Seaweed-seagrass attributes may be modified by habitat-attributes and depend on the methods used to detect impact.

*Method*. To test if the habitat and the experimental context modify impact, we extracted data on the ‘latitude’ where the experiment was conducted (a proxy for temperature and day-length combined), ‘temperature’ (<18º, 18-22º, 23-27º, >27ºC), ‘depth’, field *vs.*, laboratory conditions, and if the experiment was based on seaweed ‘removal’ or ‘addition’ methods.

*Results and discussion*. We found no effects of latitude (Fig. S1A) or depth (Fig. S1C), but impact at low temperatures were significantly larger than at high temperatures (Fig. S1B). Laboratory and addition-type experiments had significantly larger negative effects than field and removal-type experiments, respectively (Fig. S1D-E, see also discussion in manuscript). We were only able to extract crude data to test if habitat attributes modify impact. We expected that impact increases with increasing temperatures (and latitude) because Q_10_ values are higher for seaweeds than seagrasses, resulting in more stressful levels of anoxia and sulphides [1,2]. However, we found no effect of latitude but highest negative impacts at low temperatures. This result may be caused by co-varying attributes; for example, many ‘warm’ studies were conducted using attached and coenocytic seaweeds with relatively low impacts (see discussion in the paper). Of the few studies that specifically tested for effects of temperature, impacts were generally highest at high temperatures [1,2,3]. We did not find any effect of depth. However, depth co-varies with desiccation, wave regimes, disturbance levels, light levels, sedimentation, and sediment properties, and it is therefore not surprising that this result is non-significant. Only a single experiment has specifically tested if depth modified seaweed impacts, documenting a minor difference between impact recorded on 0.5 and 0.8 m depth. A few other habitat-attributes have been tested in the reviewed papers, but too few for formal meta-analysis. For example, grazing pressure can modify impact of unattached seaweeds [4] and bicarbonate and irradiance levels can modify impacts of epiphytes [5]. By contrast, nutrient addition seems not to modify impact of *Caulerpa* [6,7]. In short, many more studies are needed to better understand how local habitat conditions modify impact of seaweeds on seagrasses.

# References

1. Holmer M, Wirachwong P, Thomsen MS (2011) Negative effects of stress-resistant drift algae and high temperature on a small ephemeral seagrass species. Marine Biology 158: 297-309.

2. Höffle H, Thomsen MS, Holmer M (2011) High mortality of *Zostera marina* under high temperature regimes but minor effects of the invasive macroalgae *Gracilaria vermiculophylla*. Estuarine, Coastal and Shelf Science 92: 35-46.

3. Martinez-Luscher J, Holmer M (2010) Potential effects of the invasive species *Gracilaria vermiculophylla* on *Zostera marina* metabolism and survival. Marine Environmental Research 69: 345-349.

4. Marcia S (2000) The effects of sea urchin grazing and drift algal blooms on a subtropical seagrass bed community. Journal of Experimental Marine Biology and Ecology 246: 53-67.

5. Sand-Jensen K (1977) Effect of epiphytes on eelgrass photosynthesis. Aquatic Botany 3: 55-63.

6. Ceccherelli G, Sechi N (2002) Nutrient availability in the sediment and the reciprocal effects between the native seagrass *Cymodocea nodosa* and the introduced green alga *Caulerpa taxifolia* in a Mediterranean bay. Hydrobiologia 474: 57-66.

7. Ceccherelli G, Cinelli F (1997) Short-term effects of nutrient enrichment of the sediment and interactions between the seagrass *Cymodocea nodosa* and the introduced green alga *Caulerpa taxifolia* in a Mediterranean bay. Journal of Experimental Marine Biology and Ecology 217: 165-177.
