# Supplementary material for: A Meta-Analysis of Seaweed Impacts on Seagrasses: Generalities and Knowledge Gaps
Source: PLoS One. 2012 Jan 10;7(1):e28595. doi: 10.1371/journal.pone.0028595 (PMC3254607; doi:10.1371/journal.pone.0028595)
Supplement: Appendix S3 — Meta-analytical test results and sample sizes. (DOC) [file pone.0028595.s007.doc]

# Appendix S3. Meta-analytical test results and sample sizes

All analyses were conducted in Metawin 2.0. Figure numbers refers to the graphs as shown in the paper.

## Analysis of all 59 experiments

### Weighted analysis

Heterogeneity df Prob(Chi-Square)

Qtotal 83.3410 58 0.01628

Mean Effect Size 95% CI Bootstrap CI Bias CI

E++ -0.9566 -1.2667 to -0.6465 -1.2850 to -0.6580 -1.2834 to -0.6520

Sqrt Pooled Variance = 0.9107

Mean Study Variance = 0.9132 Ratio = 0.9973

### Un-weighted analysis

Heterogeneity df Prob(Chi-Square)

Qtotal 58.0000 58 0.47530

Mean Effect Size 95% CI Bootstrap CI Bias CI

E++ -1.4423 -1.9618 to -0.9229 -1.9792 to -0.9539 -1.9770 to -0.9500

Sqrt Pooled Variance = 1.7241

Mean Study Variance = 1.0000 Ratio = 1.7241

## Figure 1a Seaweed abundance gDWm2

Note: Not all experiments reported seaweed abundance; 17 experiments included multiple seaweed abundance levels (these were represented with >1 *d*_experiment_).

### Weighted analysis

Estimate of pooled variance: 0.7614

Predictor Value SE Prob(Norm) Prob(Rand)

Intercept -0.2640 0.2525 0.29588 0.004

Slope -0.0024 0.0009 0.01224 0.982

Model df Q Prob(Chi-Square)

Regression 1 6.2760 0.01224

Residual 44 70.4063 0.00694

Total 45 76.6823 0.00225

SUMMARY RESULTS

Heterogeneity df Prob(Chi-Square)

Qtotal 76.6823 45 0.00225

Mean Effect Size 95% CI Bootstrap CI Bias CI

E++ -0.7341 -1.0745 to -0.3936 -1.1049 to -0.4293 -1.1232 to -0.4316

Sqrt Pooled Variance = 0.8726, Mean Study Variance = 0.8867 Ratio = 0.9841

### Unweighted analysis

Estimate of pooled variance: 3.6215

Predictor Value SE Prob(Norm) Prob(Rand)

Intercept -0.2751 0.5087 0.58867 0.017

Slope -0.0040 0.0017 0.01800 0.984

Model df Q Prob(Chi-Square)

Regression 1 5.5964 0.01800

Residual 44 44.0000 0.47164

Total 45 49.5964 0.29505

SUMMARY RESULTS

Heterogeneity df Prob(Chi-Square)

Qtotal 49.5964 45 0.29505

Mean Effect Size 95% CI Bootstrap CI Bias CI

E++ -1.2164 -1.8549 to -0.5780 -1.9001 to -0.5990 -2.0238 to -0.6613

Sqrt Pooled Variance = 1.9030, Mean Study Variance = 1.0000 Ratio = 1.9030

\

## Figure 1b Seaweed abundance

Note; This analysis is based on 17 experiments that tested explicitly for impact of seaweed abundance. This test does not suffer from problems with environmental co-variation. An un-structured meta-analysis was conducted on =Δ*d* values (=Δ*d* *d*_High_ – *d*_Low_, *V* = variance of *d*).

Table: Data-set used for paired seaweed abundances.

| Exp | *d*_Low_ | *V*_Low_ | *d*_High_ | *V*_High_ | **Δ***d* | *V*_average_ |
| --- | --- | --- | --- | --- | --- | --- |
| 1 | -6.07157 | 3.738667 | -13.0371 | 14.8306 | -6.96557 | 9.284634 |
| 2 | 0.151861 | 0.335137 | -0.9814 | 0.385462 | -1.13326 | 0.360299 |
| 3 | -0.33885 | 0.677534 | -2.58581 | 1.415648 | -2.24696 | 1.046591 |
| 4 | -1.74517 | 0.974244 | -2.4691 | 1.268491 | -0.72393 | 1.121368 |
| 5 | -0.32973 | 0.679154 | -0.47656 | 0.686145 | -0.14683 | 0.682649 |
| 6 | -0.56127 | 0.701693 | -0.36209 | 0.707672 | 0.199186 | 0.704682 |
| 7 | -2.00938 | 1.310679 | -3.50647 | 3.012515 | -1.49709 | 2.161597 |
| 8 | -0.32893 | 0.686914 | -0.85388 | 0.760655 | -0.52495 | 0.723784 |
| 9 | -4.02364 | 1.739739 | -5.16306 | 2.487421 | -1.13942 | 2.11358 |
| 10 | -2.42444 | 1.928442 | -2.86816 | 3.400996 | -0.44372 | 2.664719 |
| 11 | -0.5187 | 0.690352 | -0.68442 | 0.707045 | -0.16573 | 0.698698 |
| 12 | -4.70881 | 1.799899 | -10.2698 | 8.526666 | -5.56103 | 5.163283 |
| 13 | -5.1354 | 2.012049 | -6.4758 | 2.769054 | -1.34039 | 2.390551 |
| 14 | -3.11458 | 1.038012 | -5.41374 | 2.161311 | -2.29916 | 1.599662 |
| 15 | -0.06773 | 0.766706 | -0.12453 | 1.072448 | -0.05681 | 0.919577 |
| 16 | -0.01017 | 0.688762 | 0.22056 | 0.724468 | 0.230733 | 0.706615 |
| 17 | -1.24832 | 0.82086 | -2.08888 | 1.041969 | -0.84056 | 0.931415 |

### Weighted analysis

Estimate of pooled variance: 0.0965

SUMMARY RESULTS

Heterogeneity df Prob(Chi-Square)

Qtotal 16.4525 16 0.42185

Mean Effect Size 95% CI Bootstrap CI Bias CI

E++ -0.7820 -1.3359 to -0.2280 -1.2506 to -0.4196 -1.2775 to -0.4248

Sqrt Pooled Variance = 0.3106 Mean Study Variance = 1.9573 Ratio = 0.1587

### Unweighted analysis

Estimate of pooled variance: 2.9056

SUMMARY RESULTS

Heterogeneity df Prob(Chi-Square)

Qtotal 16.0000 16 0.45296

Mean Effect Size 95% CI Bootstrap CI Bias CI

E++ -1.4503 -2.4663 to -0.4343 -2.5055 to -0.6688 -2.5748 to -0.7028

Sqrt Pooled Variance = 1.7046 Mean Study Variance = 1.0000 Ratio = 1.7046

## Figure 1c Run time (Seaweed duration)

Note: One study contributed with multiple independent *d*_experiment_ (tested explicitly for impact of sample time with independent time treatments).

### Weighted analysis

Estimate of pooled variance: 0.7397

Predictor Value SE Prob(Norm) Prob(Rand)

Intercept -1.2758 0.1804 0.00000 0.001

Slope 0.1143 0.0358 0.00143 0.001

Model df Q Prob(Chi-Square)

Regression 1 10.1636 0.00143

Residual 65 87.4591 0.03311

Total 66 97.6227 0.00692

SUMMARY RESULTS

Heterogeneity df Prob(Chi-Square)

Qtotal 97.6227 66 0.00692

Mean Effect Size 95% CI Bootstrap CI Bias CI

E++ -0.9158 -1.1967 to -0.6350 -1.2299 to -0.6301 -1.2071 to -0.6158

Sqrt Pooled Variance = 0.8601, Mean Study Variance = 0.9264 Ratio = 0.9284

### Unweighted analysis

Estimate of pooled variance: 2.5013

Predictor Value SE Prob(Norm) Prob(Rand)

Intercept -1.8772 0.2827 0.00000 0.002

Slope 0.1647 0.0590 0.00524 0.002

Model df Q Prob(Chi-Square)

Regression 1 7.7947 0.00524

Residual 65 65.0000 0.47667

Total 66 72.7947 0.26440

SUMMARY RESULTS

Heterogeneity df Prob(Chi-Square)

Qtotal 72.7947 66 0.26440

Mean Effect Size 95% CI Bootstrap CI Bias CI

E++ -1.4127 -1.8692 to -0.9563 -1.9105 to -0.9924 -1.9212 to -0.9914

Sqrt Pooled Variance = 1.5815, Mean Study Variance = 1.0000 Ratio = 1.5815

## Figure 1d Plot size (Seaweed extent)

### Weighted analysis

Estimate of pooled variance: 0.8883

Predictor Value SE Prob(Norm) Prob(Rand)

Intercept -1.0784 0.1819 0.00000 0.068

Slope 0.2561 0.2174 0.23870 0.001

Model df Q Prob(Chi-Square)

Regression 1 1.3882 0.23870

Residual 58 81.7506 0.02167

Total 59 83.1388 0.02091

SUMMARY RESULTS

Heterogeneity df Prob(Chi-Square)

Qtotal 83.1388 59 0.02091

Mean Effect Size 95% CI Bootstrap CI Bias CI

E++ -0.9715 -1.2870 to -0.6560 -1.2998 to -0.6837 -1.2802 to -0.6694

Sqrt Pooled Variance = 0.9425 , Mean Study Variance = 0.9539 Ratio = 0.9881

### Unweighted analysis

Estimate of pooled variance: 3.0087

Predictor Value SE Prob(Norm) Prob(Rand)

Intercept -1.7054 0.2952 0.00000 0.022

Slope 0.6375 0.3936 0.10527 0.022

Model df Q Prob(Chi-Square)

Regression 1 2.6238 0.10527

Residual 58 58.0000 0.47530

Total 59 60.6238 0.41706

SUMMARY RESULTS

Heterogeneity df Prob(Chi-Square)

Qtotal 60.6238 59 0.41706

Mean Effect Size 95% CI Bootstrap CI Bias CI

E++ -1.4745 -1.9918 to -0.9573 -2.0189 to -1.0438 -2.0311 to -1.0545

Sqrt Pooled Variance = 1.7346 Mean Study Variance = 1.0000 Ratio = 1.7346

## Figure 1e Seaweed origin

### Weighted analysis

Estimate of pooled variance: 0.8206

--Heterogeneity--

Class #Studies PooledVar

0 45 1.0370

1 14 0.1384

Model df Q Prob(Chi-Square) Prob(Rand)

Between 1 6.1180 0.01338 0.021

Within 57 77.6207 0.03604

Total 58 83.7386 0.01514

--Mean Effect Sizes--

Class #Studies E+ df 95% CI Bootstrap CI Bias CI

0 45 -1.1746 44 -1.5335 to -0.8157 -1.6048 to -0.8162 -1.5960 to -0.8052

1 14 -0.2910 13 -0.9600 to 0.3780 -0.6702 to 0.0899 -0.6780 to 0.0876

SUMMARY RESULTS

Heterogeneity df Prob(Chi-Square)

Qtotal 83.7386 58 0.01514

Mean Effect Size 95% CI Bootstrap CI Bias CI

E++ -0.9551 -1.2641 to -0.6460 -1.2894 to -0.6500 -1.2797 to -0.6390

Sqrt Pooled Variance = 0.9058 Mean Study Variance = 0.9132 Ratio = 0.9920

### Unweighted analysis

Estimate of pooled variance: 2.6916

--Heterogeneity--

Class #Studies PooledVar

0 45 3.5029

1 14 -0.0540

Model df Q Prob(Chi-Square) Prob(Rand)

Between 1 5.4144 0.01997 0.025

Within 57 57.0000 0.47509

Total 58 62.4144 0.32223

--Mean Effect Sizes--

Class #Studies E+ df 95% CI Bootstrap CI Bias CI

0 45 -1.7670 44 -2.3442 to -1.1897 -2.4038 to -1.2079 -2.4240 to -1.2170

1 14 -0.3988 13 -1.5081 to 0.7105 -0.9358 to 0.0748 -0.9593 to 0.0559

SUMMARY RESULTS

Heterogeneity df Prob(Chi-Square)

Qtotal 62.4144 58 0.32223

Mean Effect Size 95% CI Bootstrap CI Bias CI

E++ -1.4423 -1.9430 to -0.9416 -2.0006 to -0.9705 -2.0401 to -0.9842

Sqrt Pooled Variance = 1.6406 Mean Study Variance = 1.0000 Ratio = 1.6406

## Figure 1f Seaweed attachment

### Weighted analysis

Estimate of pooled variance: 0.8523

--Heterogeneity--

Class #Studies PooledVar

Drift 34 0.8839

Epiphyte 17 1.4006

Root 9 0.2642

Model df Q Prob(Chi-Square) Prob(Rand)

Between 2 6.9911 0.03033 0.049

Within 57 77.0573 0.03959

Total 59 84.0485 0.01779

--Mean Effect Sizes--

Class #Studies E+ df 95% CI Bootstrap CI Bias CI

Drift 34 -1.0278 33 -1.4523 to -0.6033 -1.4738 to -0.6482 -1.4694 to -0.6416

Epiphyte 17 -1.3476 16 -1.9824 to -0.7128 -2.1030 to -0.6777 -2.0592 to -0.6443

Root 9 -0.1399 8 -0.9680 to 0.6882 -0.5815 to 0.3456 -0.5924 to 0.2935

SUMMARY RESULTS

Heterogeneity df Prob(Chi-Square)

Qtotal 84.0485 59 0.01779

Mean Effect Size 95% CI Bootstrap CI Bias CI

E++ -0.9485 -1.2578 to -0.6393 -1.2926 to -0.6570 -1.2785 to -0.6442

Sqrt Pooled Variance = 0.9232 Mean Study Variance = 0.9017 Ratio = 1.0239

### Unweighted analysis

Estimate of pooled variance: 2.7571

--Heterogeneity--

Class #Studies PooledVar

Drift 34 4.2581

Epiphyte 17 1.2532

Root 9 -0.4264

Model df Q Prob(Chi-Square) Prob(Rand)

Between 2 4.9987 0.08214 0.089

Within 57 57.0000 0.47509

Total 59 61.9987 0.36969

--Mean Effect Sizes--

Class #Studies E+ df 95% CI Bootstrap CI Bias CI

Drift 34 -1.6768 33 -2.3531 to -1.0004 -2.4887 to -0.9691 -2.5513 to -1.0013

Epiphyte 17 -1.6284 16 -2.6249 to -0.6319 -2.3090 to -0.9428 -2.2484 to -0.8931

Root 9 -0.0949 8 -1.5849 to 1.3951 -0.5685 to 0.3813 -0.5564 to 0.3856

SUMMARY RESULTS

Heterogeneity df Prob(Chi-Square)

Qtotal 61.9987 59 0.36969

Mean Effect Size 95% CI Bootstrap CI Bias CI

E++ -1.4258 -1.9265 to -0.9250 -1.9091 to -0.9734 -1.9784 to -0.9901

Sqrt Pooled Variance = 1.6605 Mean Study Variance = 1.0000 Ratio = 1.6605

## Figure 1g Seaweed morphology

### Weighted analysis

Note; morphologies that only were tested in a single experiment (Articulated calcareous, Encrusting) could not be analysed.

Estimate of pooled variance: 0.7998

--Heterogeneity--

Class #Studies PooledVar

Clo 8 0.3077

Coa 12 1.2344

Fil 26 1.0010

Mix 6 0.7559

She 5 -0.1802

Model df Q Prob(Chi-Square) Prob(Rand)

Between 4 12.8388 0.01209 0.024

Within 52 71.7520 0.03606

Total 56 84.5907 0.00810

--Mean Effect Sizes--

Class #Studies E+ df 95% CI Bootstrap CI Bias CI

Clo 8 -0.1012 7 -0.9667 to 0.7644 -0.6002 to 0.3969 -0.6469 to 0.3758

Coa 12 -1.2788 11 -2.1102 to -0.4474 -2.3504 to -0.6493 -2.3792 to -0.6512

Fil 26 -1.0281 25 -1.4955 to -0.5607 -1.5539 to -0.5915 -1.5563 to -0.5911

Mix 6 -0.6207 5 -1.8896 to 0.6483 -1.5497 to 0.2431 -1.4955 to 0.3708

She 5 -2.3858 4 -3.9941 to -0.7776 -3.1163 to -1.8395 -3.1394 to -1.8656

SUMMARY RESULTS

Heterogeneity df Prob(Chi-Square)

Qtotal 84.5907 56 0.00810

Mean Effect Size 95% CI Bootstrap CI Bias CI

E++ -0.9601 -1.2731 to -0.6471 -1.2895 to -0.6587 -1.2565 to -0.6467

Sqrt Pooled Variance = 0.8943 Mean Study Variance = 0.9298 Ratio = 0.9618

### Unweighted analysis

Excluded Groups from "Test factor": Artic, Enc , Estimate of pooled variance: 2.8331

--Heterogeneity--

Class #Studies PooledVar

Clo 8 -0.3857

Coa 12 5.2940

Fil 26 3.5892

Mix 6 0.4433

She 5 -0.0401

Model df Q Prob(Chi-Square) Prob(Rand)

Between 4 7.8195 0.09842 0.116

Within 52 52.0000 0.47392

Total 56 59.8195 0.33881

--Mean Effect Sizes--

Class #Studies E+ df 95% CI Bootstrap CI Bias CI

Clo 8 -0.0316 7 -1.6684 to 1.6052 -0.5565 to 0.4608 -0.5213 to 0.5059

Coa 12 -2.0562 11 -3.3001 to -0.8124 -3.5810 to -0.8806 -3.6223 to -0.8919

Fil 26 -1.5804 25 -2.3713 to -0.7896 -2.3957 to -0.8823 -2.4944 to -0.9763

Mix 6 -0.7886 5 -2.8430 to 1.2659 -1.5785 to 0.1621 -1.6457 to 0.1209

She 5 -2.5830 4 -5.0140 to -0.1520 -3.4768 to -1.8814 -3.4768 to -1.9050

SUMMARY RESULTS

Heterogeneity df Prob(Chi-Square)

Qtotal 59.8195 56 0.33881

Mean Effect Size 95% CI Bootstrap CI Bias CI

E++ -1.4678 -1.9873 to -0.9483 -1.9914 to -1.0084 -1.9881 to -1.0051

Sqrt Pooled Variance = 1.6832 Mean Study Variance = 1.0000 Ratio = 1.6832

## Figure 1h Seaweed taxonomy (genus)

### Weighted analysis

Note; genera that only were tested in a single experiment (*Ceramium, Eucheuma, Halimeda*) could not be analyzed.

Estimate of pooled variance: 0.7658

--Heterogeneity--

Class #Studies PooledVar

Caulerpa 8 0.3077

Gracilaria 11 1.9573

Laurencia 10 0.4170

Mixed 23 1.3411

Ulva 5 -0.1802

Model df Q Prob(Chi-Square) Prob(Rand)

Between 4 16.7972 0.00212 0.005

Within 52 69.2772 0.05480

Total 56 86.0744 0.00603

--Mean Effect Sizes--

Class #Studies E+ df 95% CI Bootstrap CI Bias CI

Caulerpa 8 -0.1034 7 -0.9549 to 0.7482 -0.6132 to 0.4295 -0.6606 to 0.3711

Gracilaria 11 -1.3530 10 -2.2594 to -0.4465 -2.5655 to -0.6253 -2.7247 to -0.6805

Laurencia 10 -0.4909 9 -1.2034 to 0.2215 -1.0136 to -0.1052 -1.1343 to -0.1110

Mixed 23 -1.3069 22 -1.8400 to -0.7738 -1.8779 to -0.7656 -1.8765 to -0.7543

Ulva 5 -2.3827 4 -3.9722 to -0.7932 -3.2862 to -1.8659 -3.2862 to -1.8659

SUMMARY RESULTS

Heterogeneity df Prob(Chi-Square)

Qtotal 86.0744 56 0.00603

Mean Effect Size 95% CI Bootstrap CI Bias CI

E++ -0.9750 -1.2840 to -0.6659 -1.3143 to -0.6813 -1.3204 to -0.6857

Sqrt Pooled Variance = 0.8751 Mean Study Variance = 0.9313 Ratio = 0.9397

### Unweighted analysis

Estimate of pooled variance: 2.6117

--Heterogeneity--

Class #Studies PooledVar

Caulerpa 8 -0.3857

Gracilaria 11 5.7586

Laurencia 10 -0.2143

Mixed 23 3.7732

Ulva 5 -0.0401

Model df Q Prob(Chi-Square) Prob(Rand)

Between 4 11.2003 0.02440 0.034

Within 52 52.0000 0.47392

Total 56 63.2003 0.23711

--Mean Effect Sizes--

Class #Studies E+ df 95% CI Bootstrap CI Bias CI

Caulerpa 8 -0.0316 7 -1.6204 to 1.5572 -0.5314 to 0.4783 -0.5484 to 0.4569

Gracilaria 11 -2.1680 10 -3.4446 to -0.8913 -3.8743 to -0.8242 -3.8683 to -0.8011

Laurencia 10 -0.5135 9 -1.8731 to 0.8461 -1.1524 to -0.1083 -1.2419 to -0.1207

Mixed 23 -1.8435 22 -2.6654 to -1.0216 -2.7263 to -0.9870 -2.8994 to -1.0747

Ulva 5 -2.5830 4 -4.9427 to -0.2232 -3.4525 to -1.9171 -3.4768 to -2.0033

SUMMARY RESULTS

Heterogeneity df Prob(Chi-Square)

Qtotal 63.2003 56 0.23711

Mean Effect Size 95% CI Bootstrap CI Bias CI

E++ -1.4834 -1.9876 to -0.9791 -1.9991 to -0.9730 -2.0319 to -0.9789

Sqrt Pooled Variance = 1.6161 Mean Study Variance = 1.0000 Ratio = 1.6161

## Figure 2a Seagrass abundance (gDW/m2)

Note: Not all experiments reported seagrass abundance.

### Weighted analysis

Estimate of pooled variance: 0.9992

Predictor Value SE Prob(Norm) Prob(Rand)

Intercept -0.5090 0.2453 0.03796 0.425

Slope 0.0000 0.0002 0.83878 0.241

Model df Q Prob(Chi-Square)

Regression 1 0.0414 0.83878

Residual 46 80.2681 0.00131

Total 47 80.3094 0.00177

SUMMARY RESULTS

Heterogeneity df Prob(Chi-Square)

Qtotal 80.3094 47 0.00177

Mean Effect Size 95% CI Bootstrap CI Bias CI

E++ -0.4747 -0.8328 to -0.1166 -0.8648 to -0.1070 -0.8619 to -0.1061

Sqrt Pooled Variance = 0.9996 Mean Study Variance = 0.8893 Ratio = 1.1240

### Unweighted analysis

Estimate of pooled variance: 4.5973

Predictor Value SE Prob(Norm) Prob(Rand)

Intercept -1.0364 0.4700 0.02744 0.388

Slope 0.0001 0.0004 0.71668 0.388

Model df Q Prob(Chi-Square)

Regression 1 0.1317 0.71668

Residual 46 46.0000 0.47227

Total 47 46.1317 0.50845

SUMMARY RESULTS

Heterogeneity df Prob(Chi-Square)

Qtotal 46.1317 47 0.50845

Mean Effect Size 95% CI Bootstrap CI Bias CI

E++ -0.9192 -1.6062 to -0.2322 -1.5794 to -0.3106 -1.6342 to -0.3347

Sqrt Pooled Variance = 2.1441 Mean Study Variance = 1.0000 Ratio = 2.1441

## Figure 2b Seagrass size

Note: Experiments reporting effects on mixed seagrass species assemblages were not included.

### Weighted analysis

Estimate of pooled variance: 0.8275

--Heterogeneity--

Class #Studies PooledVar

1 7 2.1296

2 8 0.7143

3 44 0.6846

Model df Q Prob(Chi-Square) Prob(Rand)

Between 2 7.8377 0.01986 0.035

Within 56 75.5897 0.04162

Total 58 83.4274 0.01602

--Mean Effect Sizes--

Class #Studies E+ df 95% CI Bootstrap CI Bias CI

1 7 -2.0374 6 -3.2104 to -0.8644 -3.7886 to -1.1049 -3.7217 to -1.0576

2 8 -0.2744 7 -1.2493 to 0.7006 -1.3231 to 0.3558 -1.3315 to 0.3552

3 44 -0.9342 43 -1.2936 to -0.5749 -1.3167 to -0.6232 -1.3001 to -0.6129

SUMMARY RESULTS

Heterogeneity df Prob(Chi-Square)

Qtotal 83.4274 58 0.01602

Mean Effect Size 95% CI Bootstrap CI Bias CI

E++ -0.9563 -1.2661 to -0.6464 -1.3122 to -0.6764 -1.3070 to -0.6648

Sqrt Pooled Variance = 0.9097, Mean Study Variance = 0.9132 Ratio = 0.9961

### Unweighted analysis

Estimate of pooled variance: 2.6094

--Heterogeneity--

Class #Studies PooledVar

1 7 6.2048

2 8 2.2487

3 44 2.1664

Model df Q Prob(Chi-Square) Prob(Rand)

Between 2 7.8371 0.01987 0.032

Within 56 56.0000 0.47486

Total 58 63.8371 0.27886

--Mean Effect Sizes--

Class #Studies E+ df 95% CI Bootstrap CI Bias CI

1 7 -3.2490 6 -5.0062 to -1.4918 -5.0948 to -1.4888 -5.1727 to -1.5055

2 8 -0.6992 7 -2.2875 to 0.8891 -1.9903 to 0.3566 -2.0079 to 0.3400

3 44 -1.2900 43 -1.8676 to -0.7124 -1.8808 to -0.7950 -1.9780 to -0.8566

SUMMARY RESULTS

Heterogeneity df Prob(Chi-Square)

Qtotal 63.8371 58 0.27886

Mean Effect Size 95% CI Bootstrap CI Bias CI

E++ -1.4423 -1.9374 to -0.9472 -1.9615 to -0.9828 -1.9658 to -0.9837

Sqrt Pooled Variance = 1.6153 Mean Study Variance = 1.0000 Ratio = 1.6153

## Figure 2c Seagrass taxonomy (genus)

Note; genera that only were tested in a single experiment (*Enhalus*) could not be analysed.

### Weighted analysis

Estimate of pooled variance: 0.7826

--Heterogeneity--

Class #Studies PooledVar

Amp 2 -0.4560

Cym 6 -0.0139

Halod 3 2.3315

Halop 3 -0.4094

Mix 3 0.0557

Tha 17 0.2256

Zos 29 2.0644

Model df Q Prob(Chi-Square) Prob(Rand)

Between 6 35.0837 0.00000 0.001

Within 56 72.9239 0.06385

Total 62 108.0076 0.00027

--Mean Effect Sizes--

Class #Studies E+ df 95% CI Bootstrap CI Bias CI

Amp 2 0.4188 1 -9.1759 to 10.8135 -0.8019 to 0.1371 -0.8019 to 0.1371

Cym 6 -0.1631 5 -1.2615 to 0.9353 -0.5027 to 0.2653 -0.5078 to 0.2653

Halod 3 -1.3897 2 -4.1661 to 1.3867 -7.4313 to -0.1085 -3.2611 to -0.1085

Halop 3 -5.3983 2 -9.9312 to -0.8654 -7.4915 to -4.2642 -6.6708 to -4.2642

Mix 3 -1.5014 2 -4.1859 to 1.1831 -2.2721 to -0.9931 -2.2721 to -0.9931

Tha 17 -0.3971 16 -0.9295 to 0.1354 -0.7886 to -0.0829 -0.8297 to -0.1041

Zos 29 -1.3531 28 -1.8430 to -0.8632 -1.9481 to -0.7901 -1.9198 to -0.7655

SUMMARY RESULTS

Heterogeneity df Prob(Chi-Square)

Qtotal 108.0076 62 0.00027

Mean Effect Size 95% CI Bootstrap CI Bias CI

E++ -0.8981 -1.1919 to -0.6043 -1.2352 to -0.5770 -1.2207 to -0.5579

Sqrt Pooled Variance = 0.8847, Mean Study Variance = 0.9217 Ratio = 0.9598

### Unweighted analysis

Estimate of pooled variance: 2.1851

--Heterogeneity--

Class #Studies PooledVar

Amp 2 -0.9994

Cym 6 -0.6811

Halod 3 14.5023

Halop 3 1.6056

Mix 3 -0.5882

Tha 17 -0.3430

Zos 29 3.6150

Model df Q Prob(Chi-Square) Prob(Rand)

Between 6 33.9548 0.00001 0.002

Within 56 56.0000 0.47486

Total 62 89.9548 0.01169

--Mean Effect Sizes--

Class #Studies E+ df 95% CI Bootstrap CI Bias CI

Amp 2 0.4195 1 -15.2152 to 16.8542 -0.8019 to 1.371 - 0.8019 to 1.3371

Cym 6 -0.1244 5 -1.9971 to 1.7484 -0.4835 to 0.3506 -0.4640 to 0.3865

Halod 3 -2.9340 2 -7.3674 to 1.4995 -7.4313 to -0.1085 -7.4313 to -0.1085

Halop 3 -5.8537 2 -10.2872 to -1.4203 -7.4915 to -4.2642 -6.9295 to -4.2642

Mix 3 -1.6021 2 -6.0356 to 2.8313 -2.2721 to -0.9931 -2.2721 to -1.1758

Tha 17 -0.4178 16 -1.3354 to 0.4997 -0.8173 to -0.0750 -0.8395 to -0.1052

Zos 29 -1.8371 28 -2.5160 to -1.1582 -2.5940 to -1.1430 -2.6029 to -1.1429

SUMMARY RESULTS

Heterogeneity df Prob(Chi-Square)

Qtotal 89.9548 62 0.01169

Mean Effect Size 95% CI Bootstrap CI Bias CI

E++ -1.4390 -1.8885 to -0.9895 -1.9502 to -0.9418 -1.9717 to -0.9444

Sqrt Pooled Variance = 1.4782, Mean Study Variance = 1.0000 Ratio = 1.4782

## Figure S1a Temperature ranking

### Weighted analysis

Estimate of pooled variance: 0.6755

--Heterogeneity--

Class #Studies PooledVar

1 20 0.7350

2 13 0.9986

3 8 1.3656

4 18 0.3547

Model df Q Prob(Chi-Square) Prob(Rand)

Between 3 14.1761 0.00268 0.007

Within 55 76.8129 0.02764

Total 58 90.9890 0.00367

--Mean Effect Sizes--

Class #Studies E+ df 95% CI Bootstrap CI Bias CI

1 20 -1.8019 19 -2.3837 to -1.2201 -2.3418 to -1.2910 -2.3414 to -1.2887

2 13 -0.6586 12 -1.3417 to 0.0246 -1.3953 to -0.0980 -1.4091 to -0.1023

3 8 -0.8235 7 -1.7770 to 0.1299 -2.0362 to 0.0544 -2.0961 to 0.0093

4 18 -0.4897 17 -0.9850 to 0.0056 -0.9285 to -0.1397 -0.9608 to -0.1603

SUMMARY RESULTS

Heterogeneity df Prob(Chi-Square)

Qtotal 90.9890 58 0.00367

Mean Effect Size 95% CI Bootstrap CI Bias CI

E++ -0.9276 -1.2183 to -0.6368 -1.2603 to -0.6207 -1.2546 to -0.6185

Sqrt Pooled Variance = 0.8219; Mean Study Variance = 0.9132 Ratio = 0.9000

### Unweighted analysis

Estimate of pooled variance: 2.7243

--Heterogeneity--

Class #Studies PooledVar

1 20 3.3103

2 13 4.5306

3 8 3.6950

4 18 0.3947

Model df Q Prob(Chi-Square) Prob(Rand)

Between 3 6.8667 0.07627 0.088

Within 55 55.0000 0.47464

Total 58 61.8667 0.33982

--Mean Effect Sizes--

Class #Studies E+ df 95% CI Bootstrap CI Bias CI

1 20 -2.2470 19 -3.1503 to -1.3437 -3.2112 to -1.5068 -3.4025 to -1.5736

2 13 -1.3671 12 -2.5333 to -0.2010 -2.7354 to -0.3330 -2.9219 to -0.4297

3 8 -1.4314 7 -3.0448 to 0.1820 -2.9836 to -0.1340 -3.1272 to -0.2157

4 18 -0.6073 17 -1.5670 to 0.3523 -1.1949 to -0.1052 -1.2764 to -0.1384

SUMMARY RESULTS

Heterogeneity df Prob(Chi-Square)

Qtotal 61.8667 58 0.33982

Mean Effect Size 95% CI Bootstrap CI Bias CI

E++ -1.4423 -1.9453 to -0.9394 -1.9570 to -0.9465 -2.0090 to -0.9870

Sqrt Pooled Variance = 1.6505; Mean Study Variance = 1.0000 Ratio = 1.6505

## Figure S1b continuous Latitude

### Weighted analysis

Estimate of pooled variance: 0.7690

Predictor Value SE Prob(Norm) Prob(Rand)

Intercept 0.2230 0.4470 0.61798 0.001

Slope -0.0314 0.0113 0.00547 0.005

Model df Q Prob(Chi-Square)

Regression 1 7.7174 0.00547

Residual 57 78.4326 0.03141

Total 58 86.1500 0.00962

SUMMARY RESULTS

Heterogeneity df Prob(Chi-Square)

Qtotal 86.1500 58 0.00962

Mean Effect Size 95% CI Bootstrap CI Bias CI

E++ -0.9458 -1.2484 to -0.6431 -1.2680 to -0.6370 -1.2707 to -0.6414

Sqrt Pooled Variance = 0.8769; Mean Study Variance = 0.9132 Ratio = 0.9603

### Unweighted analysis

Estimate of pooled variance: 2.7663

Predictor Value SE Prob(Norm) Prob(Rand)

Intercept 0.0978 0.7949 0.90203 0.023

Slope -0.0389 0.0191 0.04099 0.023

Model df Q Prob(Chi-Square)

Regression 1 4.1764 0.04099

Residual 57 57.0000 0.47509

Total 58 61.1764 0.36265

SUMMARY RESULTS

Heterogeneity df Prob(Chi-Square)

Qtotal 61.1764 58 0.36265

Mean Effect Size 95% CI Bootstrap CI Bias CI

E++ -1.4423 -1.9481 to -0.9365 -1.9751 to -0.9787 -2.0158 to -1.0168

Sqrt Pooled Variance = 1.6632; Mean Study Variance = 1.0000 Ratio = 1.6632

## Figure S1c Depth

### Weighted analysis

Estimate of pooled variance: 0.8369

Predictor Value SE Prob(Norm) Prob(Rand)

Intercept -1.1581 0.1991 0.00000 0.020

Slope -0.2650 0.1650 0.10818 0.001

Model df Q Prob(Chi-Square)

Regression 1 2.5807 0.10818

Residual 57 80.4264 0.02219

Total 58 83.0070 0.01730

SUMMARY RESULTS

Heterogeneity df Prob(Chi-Square)

Qtotal 83.0070 58 0.01730

Mean Effect Size 95% CI Bootstrap CI Bias CI

E++ -0.9579 -1.2689 to -0.6469 -1.3146 to -0.6666 -1.3159 to -0.6658

Sqrt Pooled Variance = 0.9148, Mean Study Variance = 0.9132 Ratio = 1.0018

### Unweighted analysis

Estimate of pooled variance: 2.8337

Predictor Value SE Prob(Norm) Prob(Rand)

Intercept -1.7654 0.3141 0.00000 0.012

Slope -0.4643 0.2637 0.07823 0.012

Model df Q Prob(Chi-Square)

Regression 1 3.1014 0.07823

Residual 57 57.0000 0.47509

Total 58 60.1014 0.39953

SUMMARY RESULTS

Heterogeneity df Prob(Chi-Square)

Qtotal 60.1014 58 0.39953

Mean Effect Size 95% CI Bootstrap CI Bias CI

E++ -1.4423 -1.9526 to -0.9320 -1.9393 to -0.9659 -1.9446 to -0.9748

Sqrt Pooled Variance = 1.6834, Mean Study Variance = 1.0000 Ratio = 1.6834

## Figure S1d Experiment design; field *vs.* laboratory

### Weighted analysis

Estimate of pooled variance: 0.5898

--Heterogeneity--

Class #Studies PooledVar

field 34 0.3937

lab 26 1.4774

Model df Q Prob(Chi-Square) Prob(Rand)

Between 1 24.6911 0.00000 0.001

Within 58 73.8247 0.07865

Total 59 98.5158 0.00096

--Mean Effect Sizes--

Class #Studies E+ df 95% CI Bootstrap CI Bias CI

field 34 -0.4601 33 -0.7982 to -0.1221 -0.7820 to -0.1764 -0.7639 to -0.1568

lab 26 -1.9646 25 -2.4859 to -1.4432 -2.6307 to -1.4161 -2.6904 to -1.4325

SUMMARY RESULTS

Heterogeneity df Prob(Chi-Square)

Qtotal 98.5158 59 0.00096

Mean Effect Size 95% CI Bootstrap CI Bias CI

E++ -0.9131 -1.1910 to -0.6352 -1.2528 to -0.6163 -1.2470 to -0.6115

Sqrt Pooled Variance = 0.7680, Mean Study Variance = 0.9539 Ratio = 0.8051

### Unweighted analysis

Estimate of pooled variance: 1.9253

--Heterogeneity--

Class #Studies PooledVar

field 34 -0.0381

lab 26 4.5170

Model df Q Prob(Chi-Square) Prob(Rand)

Between 1 25.0767 0.00000 0.001

Within 58 58.0000 0.47530

Total 59 83.0767 0.02114

--Mean Effect Sizes--

Class #Studies E+ df 95% CI Bootstrap CI Bias CI

field 34 -0.5076 33 -1.1044 to 0.0892 -0.8573 to -0.1795 -0.8529 to -0.1671

lab 26 -2.7389 25 -3.4298 to -2.0481 -3.6541 to -1.9025 -3.7727 to -1.9532

SUMMARY RESULTS

Heterogeneity df Prob(Chi-Square)

Qtotal 83.0767 59 0.02114

Mean Effect Size 95% CI Bootstrap CI Bias CI

E++ -1.4745 -1.9164 to -1.0327 -2.0153 to -0.9872 -1.9651 to -0.9580

Sqrt Pooled Variance = 1.3875, Mean Study Variance = 1.0000 Ratio = 1.3875

## Figure S1e Experiment design; addition *vs.* removal

Note: A few experiments included both an addition or removal treatment (contributing with >1 d_experiment_). A = compare additions to plots without seagrass, R = compare removals to plots with seagrass, AR = compare addition plots to removal plots.

### Weighted analysis

Estimate of pooled variance: 0.7239

--Heterogeneity--

Class #Studies PooledVar

A 40 0.9883

AR 3 -0.2385

R 20 0.4083

Model df Q Prob(Chi-Square) Prob(Rand)

Between 2 16.1460 0.00031 0.005

Within 60 75.0879 0.09077

Total 62 91.2338 0.00922

--Mean Effect Sizes--

Class #Studies E+ df 95% CI Bootstrap CI Bias CI

A 40 -1.3577 39 -1.7381 to -0.9774 -1.8209 to -0.9876 -1.7669 to -0.9530

AR 3 -1.5223 2 -4.7604 to 1.7158 -2.1539 to -0.6844 -2.0990 to -0.6844

R 20 -0.1815 19 -0.6682 to 0.3052 -0.5379 to 0.1744 -0.5787 to 0.1537

SUMMARY RESULTS

Heterogeneity df Prob(Chi-Square)

Qtotal 91.2338 62 0.00922

Mean Effect Size 95% CI Bootstrap CI Bias CI

E++ -0.9155 -1.2025 to -0.6286 -1.2157 to -0.6372 -1.2140 to -0.6335

Sqrt Pooled Variance = 0.8508, Mean Study Variance = 0.8947 Ratio = 0.9510

### Unweighted analysis

Estimate of pooled variance: 2.1680

--Heterogeneity--

Class #Studies PooledVar

A 40 3.4522

AR 3 -0.3531

R 20 -0.2026

Model df Q Prob(Chi-Square) Prob(Rand)

Between 2 14.3930 0.00075 0.007

Within 60 60.0000 0.47572

Total 62 74.3930 0.13450

--Mean Effect Sizes--

Class #Studies E+ df 95% CI Bootstrap CI Bias CI

A 40 -1.9989 39 -2.5681 to -1.4296 -2.6736 to -1.4105 -2.7236 to -1.4262

AR 3 -1.6080 2 -6.0296 to 2.8135 -2.1539 to -0.6844 -2.1539 to -0.6844

R 20 -0.1525 19 -0.9856 to 0.6805 -0.5257 to 0.1976 -0.5455 to 0.1941

SUMMARY RESULTS

Heterogeneity df Prob(Chi-Square)

Qtotal 74.3930 62 0.13450

Mean Effect Size 95% CI Bootstrap CI Bias CI

E++ -1.3941 -1.8424 to -0.9458 -1.9155 to -0.9507 -1.9177 to -0.9527

Sqrt Pooled Variance = 1.4724, Mean Study Variance = 1.0000 Ratio = 1.4724
