# Supplementary material for: A Meta-Analysis of Seaweed Impacts on Seagrasses: Generalities and Knowledge Gaps
Source: PLoS One. 2012 Jan 10;7(1):e28595. doi: 10.1371/journal.pone.0028595 (PMC3254607; doi:10.1371/journal.pone.0028595)
Supplement: Appendix S4 — Publication bias. (DOC) [file pone.0028595.s008.doc]

# Appendix S4. Publication bias

**Methods**

We examined publication bias from funnel and normal quantile plots and tested for robustness of our results against potential publication bias by calculating weighted Rosenthal’s fail-safe number [1]. Bias analysis was conducted in MetaWin 2.0 [1] on the average Hedges *d* effect size per experiment (n = 59 experiments).

**Results and Discussion**

The funnel plot indicated a weak tendency for smaller sample sizes to be associated with stronger negative effects (Fig. S2). Experiment with small samples sizes had highest data variability. A normal quantile plot showed that standardized effect sizes followed normal-assumptions with no major deviations from linearity or ‘major data jumps’ suggesting that publication bias is minor. However, the rank correlation tests suggested minor publication bias (Kendall tau = 0.183, Z = 2.046, p = 0.045, Spearman Rank r^2^ = 0.323, p = 0.019). The weighted fail-safe numbers showed that 2360 non-significant studies would have to be added to the analysis to change the statistical significance of the observed main effect. This indicates that even if the publication bias against small and non-significant effects occurs, the results of our main meta-analyses is reasonable robust and unlikely to be influenced by publication bias. Also, these results suggest that studies with small sample sizes are more likely to produce extreme results than studies with larger sample sizes. We see no particular reason why some seagrass/seaweed tests attributes (see manuscript) should have a higher proportion of bias than any other, i.e., even if minor publication bias exist, this will likely be relatively similar between test attributes (and thereby not change differences between effect sizes for different test-attributes).

**References**

1. Rosenberg MS, Adams DC, Gurevitch J (2000) Metawin: Statistical software for meta-analysis. Massachusetts: Sinauer Associates. 128 p.
