# Supplementary material for: A Meta-Analysis of Seaweed Impacts on Seagrasses: Generalities and Knowledge Gaps
Source: PLoS One. 2012 Jan 10;7(1):e28595. doi: 10.1371/journal.pone.0028595 (PMC3254607; doi:10.1371/journal.pone.0028595)
Supplement: Appendix S5 — List of research gaps in seaweed-seagrass interaction studies. (DOC) [file pone.0028595.s009.doc]

# Appendix S5: List of research gaps in seaweed-seagrass interaction studies

Addressing gaps 1-12 will provide much-needed information to build conceptual frameworks and quantitative models to predict seaweed impact and counteract adverse effects. Gaps 1-6 reflect how seaweeds impact seagrasses (the main topic of this review), whereas gaps 7-12 address the important broader ecologic context of seaweed-seagrass interactions (which should be addressed in future reviews). It is important to limit co-variation issues and identify individual attributes used in any impact study (gaps 1-6).

| **Research gap** | **Reasoning and examples.** |
| --- | --- |
| 1. Data bias | Most studies have been conducted in the North Atlantic, at shallow depth, in small plots and on short time scales (Appendix S1). Such bias should be addressed to provide a representative and comprehensive understanding of seaweed impacts on seagrasses across biogeographical regions and between depth strata. For example, [1] is the only study to have tested for impact in the South West Pacific, [2] is the only to have conducted experiments deeper than 5 m, and [3] is the only to have conducted experiments in plots larger than 3 m^2^. |
| 2.Attribute types | Not a single impact attribute (Table 1) has yet been addressed adequately (cf. gaps 1, 3-6). Although tedious, this task is relatively straightforward to address. For example, impacts can be quantified between many different time periods [4] or many different plot sizes [5]. It is important to avoid seaweed loss in the long-term/large-plots, and ensure that the experiment does not enter seasons where impacts may be low due to co-varying attributes. It is also important to manipulate more seagrass attributes to understand how seagrasses respond to and persists under stress. For example, clonal integration and resource-sharing may modify impact, and it may therefore be vital to conserve large non-fragmented beds. Perhaps small and opportunistic seagrass species can adapt to localized seaweed stress by rapid re-colonization from adjacent clones whereas larger species simply may outlive the seaweed stress via translocation of resources. Experiments that manipulate resource sharing (e.g., by cutting seagrass rhizomes) should be useful to test these hypotheses. |
| 3.Attribute levels | Many experiments have been conducted with binary designs comparing a single control to a single treatment. This design conflates and confuses impact attributes (see [6], for detailed discussion). A minimum of 3 test levels (control included) are needed to identify attributes and >3 levels are needed to identify thresholds, non-linearity and to compare impact-curve-shapes. Multi-level tests are vital to model impact. See the Appendix S1 (column; “experimental design”) for list of treatment levels used in published papers. Only one study applied more >4 levels for any impact attribute [7] making it virtually impossible to compare and evaluate impact-curves. |
| 4.Modifying habitat-attributes | Seaweed and seagrass impacts attributes are modified by seagrass-habitat attributes, such as resource levels (e.g. nutrients, light, CO_2_, O_2_, space), abiotic conditions (e.g. temperature, salinity, desiccation, hydrodynamic energy, sedimentation, substrate conditions, day-length) and resident animals living in or around the seagrass habitat (e.g. grazers, filter feeders, predators). Manipulations of seagrass and seaweed attributes should therefore be crossed with manipulations of seaweed and seagrass attributes to target separate and combined effects of seaweed and seagrass attributes (see also Gap 6). Experimental data were inadequate to address modifying habitat-attributes with meta-analysis. See [8,9] for published examples on modifying effects of temperature and Appendix S2 for preliminary meta-analytical examples of temperature, depth and latitude effects. |
| 5.Attribute dynamics | Most impact attributes do not only depend on its ‘mean’ value (e.g. average seaweed abundance), but change over time. For example, the abundance of temperate seaweeds typically shows a strong seasonal pattern (a winter low), and even attributes like ‘attachment status’ can change, e.g., if an epiphyte dislodge and accumulate in the drift mat. Experiments should address these more realistic stress-scenarios by directly manipulating and controlling the dynamics of attributes (means, medians, minimums, maximums, and temporal changes) [10]. |
| 6.Attribute interactions | Attributes do not function in isolation, but interact in nature. Factorial experiments are needed to quantify such interactions between seaweed and seagrass attributes (Fig. 3), with modifying habitat-attributes, and with additional anthropogenic stressors. Only a few studies have tested for interactions between seaweed abundance and a second impact attribute (Appendix S1); for example, [11] tested if animals interact with seaweeds and [8] showed that seaweed impact depends on temperature. |
| 7.Competition mechanisms | The easy-to-identify attributes (Table 1) are associated with potentially more complex underlying impact mechanisms. Novel manipulations are needed to test *how* light, nutrients, oxygen, CO_2_, space, SH_2_, NH_4_, and allelochemicals (derived from the seaweed and seagrass) influence the competitive hierarchies. For example, seaweed associated SH_2_ [12,13] and allelochemicals [14] can be underlying causes of seagrass inhibition. It may also be that in some cases mutualism, rather than competition, occur, e.g. in the high intertidal zone to reduce photo-inhibition and desiccation, to reduce sediments instability [15], by reducing grazing pressures or by facilitating dispersal ([16]. |
| 8.Recovery trajectories | It is important also to document if and how seagrass recover following release of the seaweed stress. For example, 1.5 year after seaweeds were removed, biomass and short shoots were still only half of the non-impacted control [17]. This information is of obvious importance for managers, as recovery mechanisms may be facilitated, e.g. via protecting seedlings or planting nursery species. |
| 9.Reciprocal effects | Reciprocal impacts of the seagrass on the seaweed need to be documented. For example, seagrasses that have strong negative effects on seaweeds may only experience short-term impact before the seaweed is out-competed. Some seagrass species may also be strong seaweed facilitators by providing superior conditions for seaweed attachment or entanglement. A few studies have tested for reciprocal effects, so far documenting only minor negative effects on rooted seaweeds [2,18,19,20]. |
| 10.Ecosystem effect | To understand the full range of impacts of seaweeds, broader ecological effects should be documented. For example, seaweed may function as an alternative habitat for seagrass fauna that may be essential, substitutable, complimentary, antagonistic or inhibitory compared to the seagrass habitat [21,22]. Seaweeds impact not only seagrass performances, but also the suitability of the habitat for fauna, ecosystem metabolism and productivity; seaweeds likely affect energy and matter flows and modulate ecosystem subsidies to adjacent habitats. |
| 11. Manipulative *vs.* mensurative experiments | Manipulative experiments (small scale, short time, mechanistic understanding, and simulating early blooming effects) should be compared to supplementary analysis of mensurative data (large scale, long time, pragmatic understanding, late blooming effects). For example, it may be useful to evaluate epiphyte effects that have been studied in much more detail with mensurative designs [23,24,25]. |
| 12. Broad ecological context | Additional insight may be gained by comparing seaweed effects on different habitat formers (e.g. salt marshes, [26], seaweed forests [27,28], oyster reefs [29] and coral reefs [30,31] or weed impacts in terrestrial systems [32,33] or impacts of invasive species [6]. For example, fast growing seaweeds, terrestrial weeds and invasive species all exhibit similar boom-and-burst population dynamics (indeed, 6 of reviewed studies tested for impact on invasive seaweeds, cf. Appendix S1). |

**References**

1. Cummins SP, Roberts DE, Zimmerman KD (2004) Effects of the green macroalgae *Enteromorpha intestinalis* on macrobenthic and seagrass assemblages in a shallow coastal estuary. Marine Ecology Progress Series 266: 77-87.

2. Davis BC, Fourqurean JW (2001) Competition between the tropical alga, *Halimeda incrassata*, and the seagrass, *Thalassia testudinum*. Aquatic Botany 71: 217-232.

3. Ekloef JS, Henriksson R, Kautsky N (2006) Effects of tropical open-water seaweed farming on seagrass ecosystem structure and function. Marine Ecology Progress Series 325: 73-84.

4. Irlandi EA, Orlando BA, Biber PD (2004) Drift algae-epiphyte-seagrass interactions in a subtropical *Thalassia testudinum* meadow. marine Ecology Progress Series 279: 81-91.

5. Wernberg T, Thomsen MS, Tuya F, Kendrick GA, Stæhr PA, et al. (2010) The resilience of Australasian kelp beds decrease along a latitudinal gradient in ocean temperature. Ecology Letters 13: 685–694.

6. Thomsen MS, Wernberg T, Olden JD, Griffin JN, Silliman BR (2011) A framework to study the context-dependent impacts of marine invasions Journal of Experimental Marine Biology and Ecology 400: 322-327.

7. Hauxwell J, Cebrian J, Furlong C, Valiela I (2001) Macroalgal canopies contribute to eelgrass (*Zostera marina*) decline in temperate estuarine ecosystems. Ecology: 1007-1022.

8. Holmer M, Wirachwong P, Thomsen MS (2011) Negative effects of stress-resistant drift algae and high temperature on a small ephemeral seagrass species. Marine Biology 158: 297-309.

9. Höffle H, Thomsen MS, Holmer M (2011) High mortality of *Zostera marina* under high temperature regimes but minor effects of the invasive macroalgae *Gracilaria vermiculophylla*. Estuarine, Coastal and Shelf Science 92: 35-46.

10. Incera M, Bertocci I, Benedetti-Cecchi L (2010) Effects of mean intensity and temporal variability of disturbance on the invasion of *Caulerpa racemosa* var. cylindracea (Caulerpales) in rock pools. Biological Invasions 12: 501-514.

11. Marcia S (2000) The effects of sea urchin grazing and drift algal blooms on a subtropical seagrass bed community. Journal of Experimental Marine Biology and Ecology 246: 53-67.

12. Holmer M, Nielsen RM (2007) Effects of filamentous algal mats on sulfide invasion in eelgrass (*Zostera marina*). Journal of Experimental Marine Biology and Ecology 353: 245-252.

13. Holmer M, Frederiksen MS, Møllegaard H (2005) Sulfur accumulation in eelgrass (*Zostera marina*) and effect of sulfur on eelgrass growth. Aquatic Botany 81: 367-379.

14. Raniello R, Mollo E, Lorenti M, Gavagnin M, Buia MC (2007) Phytotoxic activity of caulerpenyne from the Mediterranean invasive variety of *Caulerpa racemosa*: a potential allelochemical. Biological Invasions 9: 361-368.

15. Williams SL (1990) Experimental studies of Caribbean seagrass bed development. Ecological Monographs 60: 449-469.

16. Holmquist JG (1994) Benthic macroalgae as a dispersal mechanism for fauna: influence of a marine tumbleweed. Journal of Experimental Marine Biology and Ecology 180: 235-251.

17. Holmquist JG (1997) Disturbance and gap formation in a marine benthic mosaic - influence of shifting macroalgal patches on seagrass structure and mobile invertebrates. Marine Ecology Progress Series 158: 121-130.

18. Ceccherelli G, Cinelli F (1997) Short-term effects of nutrient enrichment of the sediment and interactions between the seagrass *Cymodocea nodosa* and the introduced green alga *Caulerpa taxifolia* in a Mediterranean bay. Journal of Experimental Marine Biology and Ecology 217: 165-177.

19. Ceccherelli G, Sechi N (2002) Nutrient availability in the sediment and the reciprocal effects between the native seagrass *Cymodocea nodosa* and the introduced green alga *Caulerpa taxifolia* in a Mediterranean bay. Hydrobiologia 474: 57-66.

20. Taplin KA, Irlandi EA, Raves R (2005) Interference between the macroalga *Caulerpa prolifera* and the seagrass *Halodule wrightii*. Aquatic Botany 83: 175-186.

21. Thomsen MS (2010) Experimental evidence for positive effects of invasive seaweed on native invertebrates via habitat-formation in a seagrass bed. Aquatic Invasions 5: 341–346.

22. Thomsen MS, Wernberg T, Altieri AH, Tuya F, Gulbransen D, et al. (2010) Habitat Cascades: The Conceptual Context and Global Relevance of Facilitation Cascades via Habitat Formation and Modification. Integrative and Comparative Biology 50: 158-175.

23. Frankovich TA, Fourqurean JW (1997) Seagrass epiphyte loads along a nutrient availability gradient, Florida Bay, USA. Marine Ecology-Progress Series 159: 37-50.

24. Cambridge ML, How JR, Lavery PS, Vanderklift MA (2007) Retrospective analysis of epiphyte assemblages in relation to seagrass loss in a eutrophic coastal embayment. Marine Ecology Progress Series 346: 97–107.

25. Vanderklift MA, Lavery PS (2000) Patchiness in assemblages of epiphytic macroalgae on *Posidonia coriacea* at a hierarchy of spatial scales. Marine Ecology Progress Series 192: 127-135.

26. Thomsen MS, McGlathery KJ, Schwarzschild A, Silliman BR (2009) Distribution and ecological role of the non-native macroalga *Gracilaria vermiculophylla* in Virginia salt marshes. Biological Invasions 11: 2303-2316.

27. Russell BD, Elsdon TS, Gillanders BM, Connell SD (2005) Nutrients increase epiphyte loads: broad-scale observations and an experimental assessment Marine Biology 147: 551-558.

28. Worm B, Sommer U (2000) Rapid direct and indirect effects of a single nutrient pulse in a seaweed-epiphyte-grazer system. Marine Ecology Progress Series 202: 283-288.

29. Thomsen MS, McGlathery K (2006) Effects of accumulations of sediments and drift algae on recruitment of sessile organisms associated with oyster reefs. Journal of Experimental Marine Biology and Ecology 328: 22-34.

30. Rasher DB, Hay ME (2010) Chemically rich seaweeds poison corals when not controlled by herbivores. Proceedings of the National Academy of Sciences 107: 9683–9688.

31. Dudgeon SR, Aronson RB, Bruno JF, Precht WF (2010) Phase shifts and stable states on coral reefs. Marine Ecology Progress Series 413: 201-216.

32. Crosti R, Cascone C, Cipollaro S (2010) Use of a weed risk assessment for the Mediterranean region of Central Italy to prevent loss of functionality and biodiversity in agro-ecosystems. Biological Invasions 12: 1607-1616.

33. Vila M, Williamson M, Lonsdale M (2004) Competition experiments on alien weeds with crops: lessons for measuring plant invasion impact? Biological Invasions 6: 59-69.
